# Metabo-epigenetic circuits of heart failure: chromatin-modifying enzymes as determinants of metabolic plasticity

**DOI:** 10.1038/s44321-025-00343-y

**Published:** 2025-12-11

**Authors:** Mark E Pepin, Xuemin Gong, Almut Schulze, Johannes Backs

**Affiliations:** 1 Heidelberg University, Medical Faculty Heidelberg, Institute of Experimental Cardiology, 69115 Heidelberg, Germany; 2 Heidelberg University Hospital, Department of Internal Medicine VIII, 69115 Heidelberg, Germany; 3 German Center for Cardiovascular Research (DZHK), Partner Site Heidelberg/Mannheim, 69120 Heidelberg, Germany; 4 Stanford Cardiovascular Institute, Stanford University School of Medicine, Stanford, CA USA; 5 Department of Medicine, Division of Cardiovascular Medicine, Stanford University School of Medicine, Stanford, CA USA; 6 Division of Tumor Metabolism and Microenvironment, German Cancer Research Center (DKFZ) and FSPA-ZMBH Alliance, Heidelberg, Germany; 7 Molecular Medicine Partnership Unit, European Molecular Biology Laboratory (EMBL), 69117 Heidelberg, Germany; 8 Heidelberg University, 69120 Heidelberg, Germany; 9 Helmholtz Institute for Translational AngioCardioScience (HI-TAC) of the Max Delbrück Center for Molecular Medicine in the Helmholtz Association (MDC) at Heidelberg University, Heidelberg, 69117 Germany

**Keywords:** Metabolism, Epigenetics, Heart Failure, Cardiomyopathy, Signaling, Cardiovascular System, Chromatin, Transcription & Genomics, Metabolism

## Abstract

Metabolic adaptations are a functional requirement for the heart to accommodate its broad range of physiologic operating conditions. It is increasingly recognized that persistent and exaggerated metabolic alterations precede adverse cardiac remodeling leading to heart failure. These metabolic shifts are coupled with changes in cardiac gene expression, driven in part by chromatin-modifying enzymes, which have recently been identified as both sensors and transducers of metabolic stress and gene regulatory networks, respectively. This review synthesizes the latest evidence implicating chromatin-modifying enzymes as key regulators of metabolic reprogramming in the heart, providing a framework to understand how metabolic stressors are incorporated as epigenetic modifications that regulate cardiac gene expression. We propose a model of ‘metabo-epigenetic circuitry’ within which energy metabolic perturbations drive transcriptional and epigenetic changes that ultimately contribute to cardiac dysfunction. Although many nodes in these circuits remain unidentified, this viewpoint opens new avenues for investigating chromatin-modifying enzymes as therapeutic targets to halt the metabolic programs that promote heart failure.

## Abstract (web summary)

J Backs and colleagues summarize the latest evidence suggesting that chromatin-modifying enzymes are key regulators of heart metabolic reprogramming and provide a framework for understanding how metabolic stressors are incorporated as epigenetic modifications that regulate cardiac gene expression.

## Graphical abstract

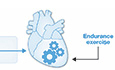

## Introduction

Compared to other tissues, the heart consumes a remarkable quantity of fuel, daily consuming over 30 kg of ATP to meet the body’s resting hemodynamic requirements (Dorn, 2013). And yet, cardiomyocytes contain only enough biochemical energy to execute roughly 10 ventricular contractions, relying on a continual influx of circulating metabolic fuels. Maintaining this dynamic equilibrium is further complicated by the heart’s wide range of operating conditions, alterations in myocardial substrate delivery, and preferential utilization of individual substrates (Costantino et al, 2019; Lopaschuk and Kelly, 2008). The regulation of cardiac metabolism therefore depends on coupling fuel consumption to the body’s ever-changing hemodynamic requirements. Pathological cardiac metabolic shifts are found in an array of clinical contexts, including familial monogenic cardiomyopathies and lifestyle-associated cardiometabolic disease (Aminian et al, 2019; Shah et al, 2020). Familial-based sequencing and genome-wide association studies have together identified over a thousand rare genetic variants that individually correlate with heart disease risk (Burke et al, 2016). However, the prognostic and therapeutic value of these genetic discoveries remains limited owing to their rarity, small effect size, variable penetrance, and unpredictable manifestations—or pleiotropy—even among monogenic etiologies (Mazzarotto et al, 2020). Heart disease risk is also highly variable in response to the most well-studied environmental and lifestyle-associated factors, such as hypertension (Rodeheffer, 2011), ischemic heart disease (Wolk et al, 1972), obesity (Alpert et al, 2014), and diabetes mellitus (Kenny and Abel, 2019). Recent studies have therefore begun to explore the molecular basis of gene-environment interactions (Smith et al, 2010; Pepin et al, 2025), or epigenetic processes, as the key determinants of heart disease susceptibility and pathogenesis.

**Figure 1:**
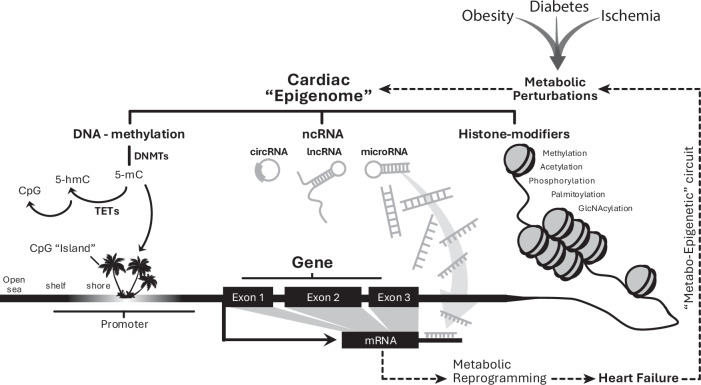
Epigenetic mechanisms as regulatory features of metabolic gene programs in heart failure. DNA methylation can be covalently added to cytosine moieties via DNA methyltransferases (DNMTs) and removed via ten-eleven translocases (TETs), with regions of methylation dynamics centered around CpG regions based on CpG content. Non-coding RNAs (ncRNA) includes long non-coding and microRNAs and classically affects mRNA stability and/or degradation post-transcriptionally. Lastly, histone-modifying enzymes covalently add a variety of functional groups to modulate transcription factor affinity and/or gene accessibility by influencing chromatin folding and macrostructure.

Although initially discovered for their role in determining cellular fate by regulating developmental programs (Waddington, 2011), epigenomic alterations are now known to encompass adult-onset diseases, and the epigenome remains responsive to both physiologic (Etchegaray and Mostoslavsky, 2016) and pathologic (Joehanes et al, 2016) stimuli. Epigenetic modifications are broadly defined as covalent modifications that occur either directly onto DNA (e.g., cytosine C-5 methylation) or to its auxiliary transcriptional structures (e.g., histone proteins) (Fig. 1). Often, post-translational regulation of mRNA stability (e.g., non-coding RNAs) is included. Although transgenerational studies of non-Mendelian traits (e.g., cardiometabolic disease) support the existence of Neo-Lamarckian inheritance patterns via epigenetic transference (Loison, 2021), this review does not delve into heredity and focuses instead on the molecular mechanisms of adult-onset disease conferred via epigenetic machinery. Specifically, we highlight the functional relevance of chromatin-modifying enzymes (i.e., DNA- and histone-modifying enzymes) that are known to influence either accessibility or activity states of genes (Bird, 2007).

**Figure 2:**
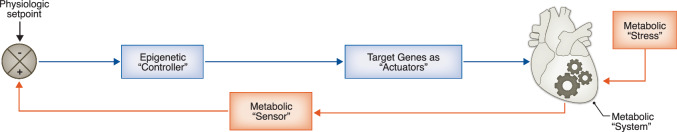
A conceptual framework of “metabo-epigenetic” signaling in the heart. Metabolic perturbations act as “sensors” that generate molecular signals influencing myocardial gene expression (“actuators”) through epigenetic regulatory mechanisms (“controllers”), forming a feedback loop analogous to a proportional–integral–derivative (PID) control system.

Emerging evidence points to the functional relevance of chromatin-modifying enzymes as both direct and indirect regulators of cardiac metabolism. Conversely, the failing heart undergoes significant epigenetic and metabolic changes including intermediary metabolites even before the onset of cardiac structural or functional impairment (Razeghi et al, 2001; Kundu et al, 2015), illustrating a conceptual framework whereby environmental perturbations leverage existing ‘metabo-epigenetic circuits’ to dysregulate cardiac function (Fig. 2).

In this review, we summarize established and emerging concepts pertaining to the role of cardiac metabolism and epigenetics in the pathogenesis of cardiomyopathy. Specifically, we highlight evidence that ‘metabo-epigenetic circuits’ integrate metabolic stressors into the transcriptional signals that influence cardiac function and how chromatin modifying enzymes regulate metabolic programs. Missing nodes emerge from within these signaling networks, the discovery of which would manifest therapeutic opportunities to rewrite the metabolic and epigenetic origins of heart disease.

## A synopsis of cardiac metabolic plasticity

Several transitions occur throughout cardiac development and into adulthood that influence metabolic signatures. The healthy adult heart exhibits a clear preference for fatty acid oxidation (FAO), yet it is not dependent on FAO to sustain baseline cardiac function. Limiting fatty acid uptake via cardiac-specific knockout of CD36 (fatty acid translocase) does not cause cardiac dysfunction (Nagendran et al, 2013). Similarly, mice lacking cardiac peroxisome proliferator-activated receptor alpha (PPARα^−/−^) exhibit normal structural and hemodynamic properties in the short-term; however, these mice eventually develop cardiac structural abnormalities reminiscent of human dilated cardiomyopathy when subjected to chronic PPARα genetic disruption in aged (9-month-old) mice (Guellich et al, 2007). It has become apparent in these studies, therefore, that the consumption of alternative fuels accommodates reduced fatty acid metabolism, balancing the heart’s cumulative metabolic demands (Kassiotis et al, 2008). Consequently, fatty acid metabolism is not required to maintain baseline cardiac function owing to the metabolic reserve that is afforded by the heart’s alternate fuel sources.

In contrast to the adult heart, the fetal heart exhibits a distinct metabolic profile and circulatory physiology. Beginning around 8 weeks of gestation, fetal circulation is maintained by parallel cardiac outputs from both ventricles through in utero shunts that bypass the pulmonary circuit (Doubilet and Benson, 1995). Owing to the low-resistance of placental circulation, fetal mean arterial pressures are significantly lower than in adults, typically ranging from 25–45 mmHg (Struijk et al, 2008), with ejection fractions between 60 and 90% (Simioni et al, 2011). Owing to low oxygen tension—attributable to the high oxygen affinity of fetal hemoglobin (Allen et al, 1953)—the fetal heart thrives on abundant glucose and lactate (Fisher, 1984; Lopaschuk et al, 1992). This preference is supported by elevated expression of key glycolytic enzymes and transporters (GLUT1, HK1, PFK1, LDHA) (Razeghi et al, 2001) and reduced expression of fatty acid oxidation genes and regulators such as CPT-1b, MCD, PGC-1α, and PPARα (Lopaschuk et al, 1992).

### Metabolic perturbations in dilated cardiomyopathy

When it fails, the heart initially compensates by reactivating a metabolic program characteristic of fetal hearts (Taegtmeyer et al, 2010; Komuro and Yazaki, 1993), increasing glycolysis and ketone body utilization (Oka et al, 2015; Doenst et al, 2013; Aubert et al, 2016). This process of metabolic substrate switching has been recognized for nearly half a century, wherein the failing adult heart increases glucose utilization for energetic supply while simultaneously lowering fatty acid oxidation (Bishop and Altschuld, 1970). The metabolic derangements of heart failure are now known to precede both the morphological changes and clinical manifestations of heart failure. Non-invasive metabolic assessments of patients with hypertension have since uncovered that, even before the onset of ventricular dysfunction, glucose utilization is augmented relative to non-hypertensive patients (Hamirani et al, 2016). Metabolomics of circulating metabolites confirmed that consumption of ketones and lactate increases in heart failure (Murashige et al, 2020). For this reason, the traditional concept that the failing heart lacks fuel for energy production (Neubauer, 2007) has lost favor, shifting instead towards an understanding that metabolic substrates employed for energy production act as regulatory intermediates to modulate cardiac performance.

### Cardiac metabolic perturbations of cardiometabolic disease

The prevalence of obesity, diabetes and heart failure has exceeded epidemic proportions across Western society (Braunwald, 1997; Mant et al, 2009; Roger, 2013), accompanied by rising rates of end-organ complications such as cardiomyopathy. While diabetes often coexists with traditional risk factors like coronary artery disease and arterial hypertension, it independently confers a fourfold increased risk of heart failure (Mahmood and Wang, 2013). Diabetic patients are at a greater risk of hospitalization and cardiovascular-related mortality (Dei Cas et al, 2015; Sarma et al, 2013), and show a diminished response to guideline-directed therapies for heart failure (HF) with reduced ejection fraction (HFrEF) (Dei Cas et al, 2015). Heart failure attributable to metabolic syndrome—often termed cardiometabolic heart failure with preserved ejection fraction (HFpEF)—typically involves features of both systolic and diastolic dysfunction (Ritchie and Abel, 2020; Zarich and Nesto, 1989). Notably, up to half of asymptomatic individuals with diabetes exhibit subclinical signs of myocardial dysfunction (Marwick et al, 2018; Boyer et al, 2004). Tighter glycemic control does not improve outcomes in HFpEF, and intensive glucose lowering may actually worsen cardiovascular outcomes (Lejeune et al, 2021; Shi et al, 2024).

Distinct from dilated cardiomyopathies, HFpEF exhibits unique metabolic and functional derangements (Maack et al, 2018). While failing hearts often increase glucose and ketone metabolism (Oka et al, 2015; Doenst et al, 2013; Aubert et al, 2016), diabetic hearts rely heavily on fatty acid oxidation, driven by insulin resistance and impaired glucose uptake (Shao and Tian, 2015; Rodrigues et al, 1995; Pepin et al, 2025). This observation has motivated functional studies seeking to understand the consequences of the diabetic heart’s persistent reliance on fatty acids. In this effort, Umbarawan et al demonstrated that impaired fatty acid uptake via FABP4/5 deficiency worsens cardiac dysfunction in diabetic mice (Umbarawan et al, 2020), while increased lipolysis through *Pnpla2* (ATGL) overexpression can be protective (Kienesberger et al, 2012, 2013). However, excessive lipid accumulation disrupts homeostatic signaling events in subcellular compartments of the mitochondrion, cytosol, and nucleus (Haemmerle et al, 2006, 2011; Wende and Abel, 2010).

Efforts to restore glucose utilization have thus far yielded mixed results. While cardiomyocyte-specific *Glut4* expression failed to improve heart function in diabetic mice (Wende et al, 2020), overexpression of *Glut1* reduced fatty acid oxidation but impaired contractile reserve (Yan et al, 2009). Additionally, Brahma et al showed that diabetic hyperglycemia suppresses ketone metabolism by inhibiting proteins 3-hydroxybutyrate dehydrogenase 1 (BDH1) and 3-oxoacid CoA-transferase 1 (OXCT1) via O-GlcNAcylation, revealing complex substrate competition in the diabetic heart (Brahma et al, 2020).

## Rare human genetic cardiomyopathies highlight metabolic origins of heart failure

Fine mapping and mechanistic validation of rare human genetic variants together reinforce a metabolic axis of dilated cardiomyopathy. Genetic contributions are estimated to account for roughly 70% of the lifetime risk for heart disease, even after adjustment for modifiable lifestyle factors and comorbid illnesses (Lee et al, 2006). Although most of these common variants affect lipid metabolism, other monogenic cardiomyopathies occur solely via loss-of-function mutations within metabolic genes (Table 1). In the following section, we summarize the mechanistic insights incidentally gained from familial monogenic dilated cardiomyopathies harboring metabolic intermediate gene mutations.

**Table 1:** Monogenic etiologies of metabolic cardiomyopathy.

Gene	Molecular function	Cardiac consequence	Clinical syndrome	Reference
MT-TL1	mt-tRNA defects → reduced OXPHOS expression	HCM, DCM	MELAS	(Hsu et al, 2016; Burattini et al, 2024)
MT-TK	Mitochondrial tRNA-Lys (mt-protein synthesis)	HCM, DCM	MERFF	(Catteruccia et al, 2015)
POLG1	Mitochondrial DNA polymerase (mtDNA replication/repair)	ACM, DCM	Mitochondrial DNA depletion syndrome (Alper’s disease)	(Spracklen et al, 2021)
ACAD9	Complex I assembly factor and acyl-CoA dehydrogenase (fatty acid β-oxidation)	DCM	ACAD9 deficiency	(Dewulf et al, 2016)
ACADVL	Very long-chain fatty acid β-oxidation	HCM, Lipid accumulation	VLCAD deficiency	(Parini et al, 1998)
AARS2	Mitochondrial alanyl-tRNA synthetase (mt-protein synthesis)	HCM	infantile mitochondrial cardiomyopathy	(Zhang et al, 2024)
COX15	Heme A biosynthesis (cytochrome c oxidase assembly, complex IV)	Early-onset fatal HCM	Leigh syndrome	(Antonicka et al, 2002)
CPT2	Carnitine palmitoyltransferase II (fatty acid transport)	HCM, Lipid accumulation	CPT2 deficiency	(Pereyra et al, 2017)
ELAC2	Mitochondrial RNase Z (tRNA 3’ end processing, mtRNA maturation)	Severe infantile-onset HCM	Combined oxidative phosphorylation deficiency 17	(Saoura et al, 2019)
ETFA/B/DH	Electron transfer flavoprotein (fatty acid/amino acid oxidation)	HCM, Lipid Accumulation	Multiple acyl-CoA dehydrogenase deficiency (MADD)	(Singla et al, 2007)
FKRP	Ribitol-5-phosphate transferase (glycosylation of α-dystroglycan)	DCM	FKRP-CDG (dystroglycanopathy)	(Libell et al, 2020)
HADHA	Long-chain 3-hydroxyacyl-CoA dehydrogenase (fatty acid β-oxidation)	HCM, DCM	LCHAD/Trifunctional protein deficiency	(Gaston et al, 2023)
GLA	α-galactosidase A (lysosomal glycosphingolipid degradation)	HCM	Fabry disease	(Weissman et al, 2024)
LAMP2	Lysosomal-associated membrane protein 2 (autophagy)	HCM	Danon disease	(Nishino et al, 2000)
GAA	Acid α-glucosidase (glycogen hydrolysis)	HCM	Pompe disease (glycogenosis type II)	(Taverna et al, 2020)
PRKAG2	AMP-activated protein kinase γ2 (energy sensing)	HCM	PRKAG2 syndrome	(Ahamed et al, 2020)
AGK	Acylglycerol kinase (phospholipid metabolism)	HCM	Sengers syndrome	(Das et al, 2019)
DPM3	Dolichol-phosphate mannosyltransferase (N-glycosylation)	DCM	Congenital disorder of glycosylation (MDDGC15)	(Svahn et al, 2019)
PNPLA2	Hydrolyzes cytoplasmic triacylglycerol (TAG) in lipid droplets (lipolysis)	DCM, HCM	Neutral lipid storage disease with myopathy (NLSDM)	(Wang et al, 2024)
ABHD5	Serine protease (links lipid and glucose metabolism via HDAC4-NT production)	DCM	Chanarin-Dorfman Syndrome (CDS)	(Mangukiya et al, 2023)

Variants within metabolic genes known to cause cardiopathy are listed, along with the molecular function and clinical syndrome.

*ACM* arrhythmogenic cardiomyopathy; *HCM* hypertrophic cardiomyopathy;* DCM* dilated cardiomyopathy; *MELAS* mitochondrial encephalomyopathy, lactic acidosis, and stroke-like episodes; *MERFF* myoclonus epilepsy with ragged-red fibers.

Pathogenic variants associated with *PNPLA2* (well known as ATGL), the enzyme that catalyzes lipolysis to hydrolyze TAGs to DAGs to liberate intracellular lipid droplets (Smirnova et al, 2006), produce a severe cardiac phenotype leading to heart failure, termed neutral lipid storage disease with cardiomyopathy (NLSD-CM) (Fischer et al, 2007; Schweiger et al, 2009; Pennisi et al, 2017). The clinical manifestations of NLSD-CM depend on the type of mutation and on residual enzymatic activity (Schweiger et al, 2009), but it has also been theorized to occur from alterations in chromatin accessibility (Pennisi et al, 2017). The clinical phenotype of human NLSD-CM resembles that of *Pnpla2*-deficient mice, wherein cardiac lipid droplet accumulation and reduced fatty acid oxidation (FAO) accompany progressive cardiac dysfunction and eventually decompensated heart failure (Haemmerle et al, 2006, 2011). In mice, ventricular systolic function can be corrected by both overexpressing *Pnpla2* and treatment with PPARα agonist (Haemmerle et al, 2011).

As a coactivator of PNPLA2, ABHD5 (well known as CGI-58) has also been shown to produce similar phenotypic manifestations when genetically disrupted in mice, although far fewer clinical cases of genetic *ABHD5* mutations have been found (Pennisi et al, 2017). Constitutive and cardiomyocyte-specific knockout of *Abhd5* in mice produces a severe cardiomyopathy (Jebessa et al, 2019). The latter study revealed that—other than FAO and lipotoxicity—a tandem loss of HDAC4-NT (an N-terminal proteolytic fragment of histone deacetylase 4, which is produced by a yet undiscovered proteolytic activity of Abhd5) accounts for the functional phenotype in mice. Mechanistically, the loss of HDAC4-NT activates a distinct transcriptional program via transcription factor Myocyte Enhancer Factor 2 (MEF2) that activates nuclear receptor subfamily 4 group A member (NR4A1), a key enzyme that activates the hexosamine biosynthetic pathway (HBP) and subsequent protein O-GlcNAcylation of calcium handling proteins, in particular a specific splicing variant of stromal interaction molecule 1 (STIM1-L) (Lehmann et al, 2018). Thus, the discovery that HDAC4-NT, which itself is sufficient to rescue cardiac dysfunction by cardiac gene therapy in *Abhd5*-deficient mice despite persisting lipid droplet accumulation, challenges the concept of lipotoxicity and directs the attention to pathological accumulation of intermediates and byproducts of glucose metabolism as drivers of transcriptionally-driven cardiac dysfunction. HDAC4-NT critically regulates the production of these glycolytic byproducts. This finding necessitates further studies that weigh the relative contributions of lipotoxicity versus glucotoxicity in both NLSD-CM and other, more common cardiometabolic etiologies of heart failure.

## Metabo-epigenetic sensors

Metabolic intermediates serve not only as fuels but also as signaling molecules that influence chromatin-modifying enzymatic activity. In 1924, Otto Warburg first reported that cancer cells prefer anaerobic glycolytic metabolism, or “fermentation,” even when exposed to oxygen-rich environments, a phenomenon that later became coined as the Warburg Effect (Warburg, 1956). Many of the metabolic alterations that are characteristic of malignancies, such as changes in TCA cycle flux and substrate preference, mirror those found in the failing heart (Taegtmeyer et al, 2017). In this manner, an array of metabolic intermediates and byproducts collectively defined as “oncometabolites” (Yang et al, 2013) have also been studied as signaling molecules that promote heart failure pathogenesis.

In non-cardiac tissue, TCA cycle intermediates including succinate and fumarate have been shown to influence chromatin-modifying enzymes. Mitochondrial accumulation of both succinate and fumarate via loss-of-function mutations of succinate dehydrogenase (SDH) or fumarate hydratase (FH) blocks the activity of ten-eleven translocase (TET) dioxygenases to inhibit nuclear DNA demethylation and JmjC domain-containing histone demethylases (KDM), thus de-repressing tumor suppressor and metabolic genes (Xiao et al, 2012). Similarly, gain-of-function mutations in tumor-derived isoforms of isocitrate dehydrogenase (IDH1 and IDH2) drive the accumulation of D-2-hydroxyglutarate, a competitive TET and KDM inhibitor, and blocks both histone demethylation and 5-hydroxymethylcytosine oxidation (Waitkus et al, 2018; Xu et al, 2011; Chowdhury et al, 2011).

In the heart, expression of mutant IDH2 in mice leads to D-2-hydroxyglutarate accumulation, impaired oxidative capacity, and cardiomyopathy (Karlstaedt et al, 2016; Akbay et al, 2014). These metabolic changes are accompanied by alterations in histone acetylation and methylation, supporting a causal role for metabolic-epigenetic coupling in cardiac dysfunction (Taegtmeyer et al, 2017). Thus, although not fully characterized in the heart, these findings support the framework that metabolic intermediates reprogram gene expression by altering modifiers of histone and DNA methylation.

Beyond the TCA cycle, the products of glucose metabolism also confer epigenetic control of gene expression via protein O-GlcNAcylation (see also above in the section “Rare human genetic cardiomyopathies highlight metabolic origins of heart failure”). O-GlcNAcylation is known to affect tumor behavior by enhancing HDAC1 activity (Zhu et al, 2012) and modifying signaling pathways that drive malignancy (Chu et al, 2020; Wu et al, 2020). In the heart, elevated O-GlcNAcylation has been implicated in diabetic cardiomyopathy and arrhythmogenesis (Jin et al, 2020), though its effects appear context-dependent (Lunde et al, 2012; Zhu et al, 2019). A recent study demonstrated that mitochondrial dislocation of hexokinase 1 (HK1) in endothelial cells promotes its interaction with O-linked N-acetylglucosamine transferase (OGT), increasing protein O-GlcNAcylation and driving HFpEF pathogenesis (Tatekoshi et al, 2025). Deletion of the mitochondrial binding domain in HK1 led to spontaneous HFpEF with impaired angiogenesis, while reversal of O-GlcNAcylation restored vascular and diastolic function.

Taken together, these observations underscore O-GlcNAcylation as a metabolically sensitive epigenetic moiety. The modification of histone deacetylases by O-GlcNAc, particularly protein O-GlcNAcylation at serine 642 of HDAC4 (see more details below in the section “HDACs”), is unmasked to protect against maladaptive signaling through CAMKII in diabetic hearts (Kronlage et al, 2019). Given the association of protein O-GlcNAcylation with both adaptive and maladaptive cardiac remodeling (Dubois-Deruy et al, 2015; Lunde et al, 2012; Muthusamy et al, 2014), additional mechanistic studies are necessary to dissect the site-specific and context-dependent effects of this modification to determine its role in heart failure pathogenesis.

## Known metabo-epigenetic circuits

The interface between metabolism and epigenetic regulation is increasingly recognized as a contributor to heart failure pathogenesis. Below, we summarize the current evidence supporting chromatin modifiers (specifically histone acetylation and DNA methylation) as regulatory links between nutrient sensing and transcriptional control of metabolic intermediate genes. These metabo-epigenetic circuits offer insight into how metabolic states are encoded at the chromatin level, though many remain incomplete, lacking defined sensors, regulators, or target genes (Table 2), and unmask new and promising therapeutic targets.

**Table 2:** Summary table of known metabo-epigenetic circuits.

Stress	Sensor	Controller	Gene program	System variable	Species	Reference(s)
Glucose handling						
Exercise	↑ PKA / ↑ ABHD5	↑ HDAC4-NT	↓ *Nr4a1*	↓ HBP, ↑ Contractility	Mouse	(Lehmann et al, 2018)
Pressure-Overload	↑ CAMK2D	↓ HDAC4-NT	↑ *Nr4a1* → ↑*Gfpt2*	↑ HBP → ↑ STIM1L O-GlcNAc ↓ Contractility	Mouse	(Lehmann et al, 2018)
Pressure-Overload	↓ ABHD5	↓ HDAC4-NT	↑ *Nr4a1* → ↑*Gfpt2* ↑ *Pdk4* → ↑PDH-phos.	↓ Glycolysis, ↑ HBP (O-GlcNAc) ↓ Contractility	Mouse	(Jebessa et al, 2019)
Diabetes (STZ, Db/Db)	↑ HBP (O-glcNAc)	↑ HDAC4-NT	↓ *Nr4a1*	↓ HBP, ↑ Contractility	Mouse	(Kronlage et al, 2019)
–	–	↓ DNMT3A	↓ MYH7/MYH6 ↑ PPARγ	↓ Glycolysis, Lipid accumulation	In Vitro (iPSCs)	(Madsen et al, 2020, 2021)
Diabetes (STZ)	N/A	N/A	“stable”	N/A	Mouse	(Lother et al, 2020)
ICM	–	↑ miRNA-320	↓ *Pfk*, ↓ *Hsp20*	↑ Glycolysis ↓ Infarct Size	Human & Mouse	(Ren et al, 2009)
ICM	–	↑ EZH2	↑ *Pfkfb3*, ↓ *Klf15*, ↓ *Acadvl*/m/sb ↓ *Idh3a*, ↓ *Sdhb*, ↓ *Cox5a*/*b*	↑ Glycolysis ↓ FAO	In vitro & Human	(Pepin et al, 2019b)
Oxidative phosphorylation						
Pressure- Overload	IDH1/2 → D-2-hydroxy- glutarate	Histone 3 KDMs/HATs	–	↓ OX-PHOS	Mouse	(Karlstaedt et al, 2016)
DCM	–	↑DNMT3A ↓GADD45B ↑HDAC4/7	↓NRF1 target genes, ↓*Acsl5*, ↓*Acsl1*, ↓*Hadha*, ↓*Ndufa5*, ↑*Pfkfb3*, ↑*Eno2*	↑ Glycolysis ↓ FAO	In vitro (H9c2)	(Pepin et al, 2019a)
Diabetes (db/db)	–	miRNA-320	↑*Ago2* → ↑*Cd36*	↑ Fatty Acid Uptake	Mouse	(Li et al, 2019)
**Circuits controlling different aspects of cardiac functions**						
Dietary Cholesterol	–	miRNA-25	↑ *Nox4*	↑ Diastolic Dysfunction	Mouse	(Varga et al, 2013)
DCM	–	–	↑ AMOTL2 ↑ ARHGAP2 ↓ PECAM1	↑ Angiogenesis	Human	(Movassagh et al, 2010)
–	–	Sirt6^+/-^	FoxO1 →↑ *Pdk4* ↓ *Pdh*	↓ Oxygen Consumption	Mouse	(Khan et al, 2018)
–	–	Hdac3^-\-^	↑ FA Uptake, ↑ FAO, ↑ OX-PHOS, ↑ *Pparα*	Cardiac Hypertrophy, Lipid Accum.	Mouse	(Montgomery et al, 2008)

### HDACs

Histone deacetylases (HDACs) are key regulators of cardiac metabolism that link gene accessibility to metabolic gene expression to control myocardial energy homeostasis. Traditionally recognized for their roles in chromatin compaction and transcriptional repression, HDACs are now appreciated for their dynamic influence on myocardial metabolic flexibility, substrate preferences, and heart failure pathogenesis.

Acetyl-CoA, generated from glucose, glutamine or fatty acids via ATP-citrate lyase (ACL), from either pyruvate by pyruvate dehydrogenase (PDH) or acetate via acetyl-CoA synthetase (ACSS), provides the substrate for histone acetylation and thus provides an important link between energy metabolism and chromatin regulation (Wellen and Thompson, 2012; Rathmell and Newgard, 2009). In a non-cardiac (HCT116) colorectal carcinoma cell line, high-glucose media increases histone acetylation, whereas reduction in acetyl-CoA synthesis by blocking ACLY results in rapid histone deacetylation (Wellen et al, 2009). Although supplementation with fatty acids complements the cells’ bioenergetic needs during glucose deprivation, no measurable effect is observed on histone acetylation (Wellen et al, 2009). Similarly, the acetylation of several metabolic intermediate proteins (e.g., GLUT4, HK2, PFK1 and IDHA) in differentiated adipocytes is regulated in an ACL-dependent manner in accordance with glucose availability (Wellen et al, 2009).

Although global HDAC inhibition (panHDACi) mitigates the pathological features of heart failure in mice (Travers et al, 2021), class I and class II HDACs seem to control this regulatory network through distinct—yet overlapping—mechanisms, modulating transcription factor activity, metabolic enzyme expression, and post-translational modifications in a context-dependent manner. The following section explores the roles of individual HDAC isoforms as metabo-epigenetic “controllers” in the heart, showcasing their diverse contributions to cardiac function and metabolic remodeling in heart failure.

#### Class I

Among class I HDACs, HDAC3 has been identified to maintain cardiac energy metabolism (Montgomery et al, 2008). *Hdac3*-deficiency in mice leads to massive cardiac hypertrophy along with upregulation of genes associated with fatty acid uptake, fatty acid oxidation, and oxidative phosphorylation, resulting in fatty acid-induced myocardial lipid accumulation and elevated triglyceride levels (Paluvai et al, 2023). As a potential underlying mechanism, excessive activity of the nuclear receptor Peroxisome proliferator-activated receptor alpha (PPARα) is seen, placing HDAC3 within this metabo-epigenetic circuit. Because this phenotype differs from other class I HDAC knockout phenotypes, a unique role for HDAC3 in the maintenance of cardiac function and regulation of myocardial energy metabolism was assumed until only recently. However, given the recently identified phenotypic overlap of murine *Hdac4* and *Hdac3*-deficiency in the heart, it will be interesting to investigate the functional relationship of HDAC3 and HDAC4 to regulate cardiac metabolism. This is of particular interest because it is known for more than two decades that these HDACs physically interact via the transcriptional corepressor N-CoR/SMRT (Fischle et al, 2002), and thus may jointly regulate cardiac metabolism with HDAC4 serving as the signal-responsive partner.

#### Class IIa and class IIb

Class II HDACs consist of class IIa (HDAC4, HDAC5, HDAC7 and HDAC9) and class IIb HDACs (HDAC6 and HDAC10). Among these, it has been long recognized that class IIa HDAC enzymes participate in cardiac metabolic homeostasis (Zhang et al, 2002; Backs et al, 2006; Chang et al, 2004; Hsu et al, 2020; Schuster et al, 2015). These studies have shown that, in response to pressure overload, class IIa HDACs act as signal-responsive regulators of cardiac growth via non-enzymatic mechanisms resulting in MEF2 inhibition (McKinsey et al, 2000; Zhang et al, 2002; Backs et al, 2006; Chang et al, 2004). MEF2 tightly controls the expression of NR4A1 (Youn et al, 1999), which has been shown to both regulate the expression of several metabolic enzymes (Pearen and Muscat, 2010) and specifically increase the activity of the HBP (Lehmann et al, 2018).

**Figure 3:**
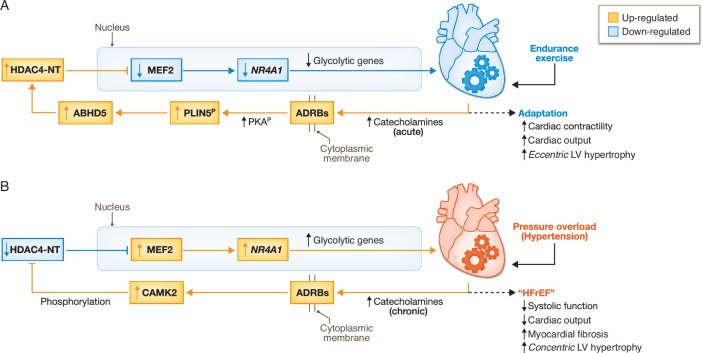
Metabo-epigenetic circuits involving HDAC4. (**A**) Physiologic response to intermittent catecholamine exposure (e.g., endurance exercise), promoting adrenergic signaling-mediated enhancement of nuclear HDAC4-NT suppression of MEF2, facilitating an adaptive metabolic response. (**B**) chronic catecholamine signaling (e.g., pressure overload, hypertension) drives pathological metabolic remodeling via CaMK2-mediated suppression of HDAC4 translocation causing de-repression of glycolytic genes.

Our prior work has highlighted a decisive role of HDAC4 via *Nr4a1* regulation as part of a transcriptional program that leads to impaired cardiac calcium homeostasis leading to systolic dysfunction (Lehmann et al, 2018) (Fig. 3). The HDAC4-MEF2-NR4A1 pathway is controlled by the proteolytic activity of the lipid droplet-associated protein ABHD5. Specifically, ABHD5 is downregulated in response to transaortic constriction (TAC) along with the product of its proteolytic activity: HDAC4-NT, leading to MEF2-dependent stimulation of inefficient cardiac glycolysis with accumulation of UDP-GlcNAc and subsequent protein O-GlcNAcylation and impaired cardiac performance (Jebessa et al, 2019).

Among the Class IIb family of HDAC enzymes, HDAC6 has attracted the most attention as a pharmaceutical candidate since its selective inhibition (e.g., via TYA-018/TN-301) lowers diastolic dysfunction in a dual high-fat diet and transaortic constriction model of HFpEF (Ranjbarvaziri et al, 2024). HDAC6 inhibition is also associated with enhanced post-translational acetylation of microtubule filaments and the sarcomeric constituent titin, which is thought to stabilize cytoskeletal integrity, reduce inflammatory signaling, and improve mitochondrial function (Lin et al, 2022). These data are puzzling, however, since global *Hdac*6-deficient (*Hdac6*-KO) mice develop worsening diastolic dysfunction (Lin et al, 2022), suggesting that baseline HDAC6 activity may be essential to maintain normal cardiac compliance. Future studies must define the dose-dependent cellular and molecular mechanisms responsible to resolve this apparent contradiction. Conditional *Hdac6* knockout studies may also be useful to identify the responsible cell type using in vivo models, and domain-specific perturbations are needed to differentiate between enzymatic and non-enzymatic functions of HDAC6. Moreover, as tubulin deacetylation has been suggested as the target of HDAC6, functional studies should gain a better understanding of HDAC6 in HFpEF regarding how tubulin deacetylation mitigates diastolic dysfunction in HFpEF. Thus, while HDAC6 inhibition shows therapeutic potential, defining its potential role in HFpEF pathogenesis warrants further study of the cell-type specific, dose-dependent, and context specific regulatory balance of epigenetic regulation and metabolic signaling.

#### Class III (sirtuins)

Sirtuins (SIRTs) comprise a family of seven NAD⁺-dependent deacetylases, among which several are heralded as epigenetic controllers of cardiac metabolic substrate sensing and gene regulation. Specifically, SIRT1, SIRT3, and SIRT6 play distinct yet interconnected roles in modulating myocardial substrate preferences, orchestrating a balance between glucose, fatty acid, and ketone metabolism.

SIRT1 is mostly found in the nucleus and regulates cardiac energy metabolism through dynamic interactions with—and deacetylation of—the transcription factors PPARα, Peroxisome Proliferator-Activated Receptor Gamma Coactivator 1-alpha (PGC-1α), and AMP-activated protein kinase (AMPK) to indirectly regulate mitochondrial biogenesis, FAO, and glucose utilization (Zhou et al, 2024). In diabetic cardiomyopathy (clinically termed cardiometabolic HFpEF), recombinant SIRT1 supplementation improves myocardial function by suppressing PPARγ signaling, reducing lipid accumulation, and downregulating genes involved in lipid trafficking and inflammation (Majeed et al, 2021). Its activation enhances oxidative metabolism (Rodgers et al, 2005), whereas reduction of SIRT1 promotes the development of dilated cardiomyopathy (Gorski et al, 2019; Prola et al, 2017).

Unlike other SIRT members, SIRT3 localizes to the mitochondrial matrix and functions in situ as a mitochondrial stress sensor (Chen et al, 2021). It post-translationally deacetylates—and thus activates—enzymes essential for FAO, ketogenesis, and oxidative phosphorylation to promote efficient ATP production and redox homeostasis (Chen et al, 2015). Conversely, *Sirt3* deficiency accelerates lipotoxicity, oxidative stress, and energetic failure. In diabetic and aging hearts, the downregulation of *Sirt3* is associated with impaired metabolic flexibility and heart failure progression; its restoration normalizes FAO and ketone metabolism to mitigate cardiac hypertrophy and fibrosis (Chen et al, 2021; Palomer et al, 2020). Similarly, nutritive supplementation with the NAD+ precursor nicotinamide riboside may mitigate diastolic dysfunction and cardiomyocyte senescence in HFpEF (Tong et al, 2021). However, recent data have challenged the central role of SIRT3 in conferring a cardioprotective role of NAD+, since nutritive restoration of NAD+ also appears to be effective in *Sirt3*-knockout mice. Specifically, by comparing the relative effects of supplementation with nicotinamide riboside in mice subjected to pressure-overload induced cardiomyopathy, Walker et al found that there were similar benefits on NAD(H) redox-sensitive short-chain dehydrogenase/reductase function and restoring mitochondrial oxidative metabolism in both *Sirt3*-deficient and wild-type mice (Walker et al, 2023). Additional studies are thus needed to clearly define the SIRT3-dependent and independent mechanisms of the NAD-associated therapeutic benefits in HFpEF.

SIRT6 acts within the nucleus to regulate expression of genes encoding lipid handling and inflammation, particularly in the diabetic heart. SIRT6 suppresses transcription of lipid transporters such as CD36 and FABP4 by occupying the DNA-binding domain of PPARγ and preventing its binding to its target promoters (Khan et al, 2021), thereby reducing fatty acid uptake and preventing lipotoxic remodeling (Wu et al, 2022). Pharmacologic activation (e.g., MDL-800) has shown promise in preclinical models of HFpEF by restoring lipid homeostasis and reducing myocardial inflammation (Wu et al, 2022).

Together, these studies support the theory that sirtuins serve as key regulators within metabo-epigenetic circuits to link nutrient availability, chromatin remodeling, and cardiac metabolic gene programming in both adaptive and pathological contexts.

### DNA methylation modifiers

Our group and others have shown that metabolic substrate switching in human HFrEF is encoded by alterations in DNA methylation. Genome-wide methylation profiling of failing human cardiac biopsies uncovered hypermethylation of oxidative metabolic gene promoters that is coupled to hypomethylation of glycolytic gene promoters, collectively recapitulating a fetal-like metabolic return of glycolytic energy metabolic preference (Pepin et al, 2019a). DNA methyltransferase 3A (DNMT3A) in human induced pluripotent stem cell-derived cardiomyocytes (iPSC-CMs) is essential for maintaining metabolic homeostasis in human cardiomyocytes (Madsen et al, 2020), while genetic overexpression of *DNMT3A* in rat cardiomyoblasts suppresses expression of gene intermediates including *ACSL1* and *HADHA* (Pepin et al, 2019a). Conversely, *DNMT3A* knockout in human engineered heart tissue causes myocardial lipid accumulation via PPARγ activation and impaired glucose metabolism due to destabilization of HIF-1α. Together, these studies highlight a causal link of de novo DNA methyltransferase activity in coordinating both structural and metabolic integrity of differentiated cardiomyocytes (Madsen et al, 2021).

The oxidative reaction of actively removing DNA methyl groups is catalyzed by ten-eleven translocation (TET) enzymes, which have similarly been linked to myocardial metabolic plasticity. TET deficiency disrupts cardiac development by inducing hyperactivation of WNT signaling and suppressing NKX2-5, a transcription factor that is essential for cardiomyocyte lineage commitment (Lan et al, 2021). Clinically, DNA methylation changes correlate with suppressed mitochondrial gene expression and enhanced glycolysis in HF patients (Pepin et al, 2019a). Mouse models of *Tet2*-deficient clonal hematopoiesis exhibit accelerated HF progression due to IL-1β-mediated inflammation (Sano et al, 2018), suggesting that hematopoietic cell-based epigenetic changes indirectly alter myocardial metabolic homeostasis. Together, these studies define DNA methylation as a metabo-epigenetic circuit, providing preliminary insights into how metabolic stress, developmental programs, and inflammation are encoded by methylome-based metabo-epigenetic regulatory gene programs.

Regarding the impact of the diabetic milieu on cardiac DNA methylation, a recent study has suggested that, although cardiac gene expression is altered in streptozotocin-induced diabetic mice, cardiac DNA methylation appears “stable,” or unaffected (Lother et al, 2020). This finding falls contrary to candidate gene studies that highlight differential DNA methylation as a measurable stress signal in diabetes (Liu et al, 2020; Chen et al, 2019). Ultimately, sufficiently powered studies are required to understand whether differential DNA methylation occurs due to the direct metabolic stress of diabetes in the heart.

## Myocardial pharmaco-epigenetic basis of metabolic therapies

Owing to the central importance of metabolic derangements in the development of heart disease, it is sensible to theorize that targeting these metabolic disruptions would remedy the hemodynamic and cardiac functional consequences that culminate into clinical heart failure (Koutroumpakis et al, 2020). Although precision-based therapies have not yet yielded a pharmacologic treatment designed to alter cardiac metabolism, several existing cardioprotective agents influence cardiac metabolism. Additionally, the effectiveness of any targeted epigenetic therapy is likely affected by guideline-directed medical therapies (GDMT) for heart failure, which themselves influence metabolic gene accessibility. For this reason, below we highlight the current understanding regarding the metabolic and epigenetic effects of existing HF therapies. It is worth highlighting that, while early evidence supports a clinical benefit, their effect on the cardiac epigenome has not yet been proven as the underlying cardioprotective mechanism.

### Sodium-glucose cotransporter-2 inhibitors (SGLT2i’s)

Two classes of metabolic therapies have been incidentally found to confer cardioprotective benefits, each originally developed to improve glycemic control for individuals with type 2 diabetes. Sodium-glucose cotransporter-2 inhibitors (SGLT2i’s) were first identified as cardioprotective agents as a result of FDA-mandated cardiovascular outcomes trials (CVOTs), which found reductions in cardiovascular endpoints independent of their glucose-lowering effects (Anker et al, 2021; Fitchett et al, 2016; Fathi et al, 2021). Although inhibition of the SGLT2 transporter promotes a mild diuresis via the osmotic effects of glycosuria, this effect confers trivial hemodynamic benefits that does not account for their cardiovascular benefits among patients with HF, especially since other diuretics have failed to demonstrate a similar benefit (Lopaschuk and Verma, 2020). The ventricular myocardium does not express SGLT2, prompting investigators to search for off target—and indirect—mechanisms of SGLT2i cardioprotection. Among these, in vitro studies demonstrated that the cardiomyocyte sodium-hydrogen exchanger (NHE-1) is inhibited by SGLT2i’s, indirectly regulating intracellular calcium handling within isolated rabbit ventricular myocytes (Baartscheer et al, 2016).

SGLT2i’s are known to exert epigenetic influence via class IIa and III HDACs. Dapagliflozin lowered *Camk2* expression in a rat model of isoproterenol-induced cardiomyopathy (Liu et al, 2023), which our group and others have shown to promote metabolic switching via nuclear exclusion of HDAC4 (Helmstadter et al, 2021; Lehmann et al, 2018). Another study used primary isolated rat fibroblast to show that treatment with either dapagliflozin or empagliflozin prevented the reduction of *Hdac6* expression caused by Angiotensin II and TGFβ exposure to lower collagen expression in cardiac fibroblasts (Ma et al, 2024). Similarly, SIRT1 is required for the cardioprotective effects of dapagliflozin in response to the transaortic constriction model of pressure overload-induced heart failure (Ren et al, 2022).

Regarding the upstream signaling that may confer these epigenetic alterations, a pre-print report found that SGLT2i activates by an off target mechanism pantothenate kinase 1 (PANK1), the rate-limiting enzyme that produces the CoA moiety from pantothenate, to stimulate metabolic flux in myocardial tissue from human subjects with heart failure (Forelli et al, 2024). Together, these data support that SGLT2 inhibition may promote epigenetic programming and/or myocardial metabolism.

### Incretin mimetic therapies (GLP-1RA’s)

The second major cardiometabolic drug class identified through CVOTs comprises the family-wide effect of glucagon-like peptide-1 receptor agonists (GLP-1RAs), termed incretin mimetics, with landmark studies including LEADER (Liraglutide) (Marso et al, 2016b), SUSTAIN-6 (Semaglutide) (Marso et al, 2016a), REWIND (Dulaglutide) (Gerstein et al, 2019), and EXSCEL (Exenatide) (Holman et al, 2017). These agents confer significant cardiovascular protection, though their direct epigenetic effects on ventricular cardiomyocytes are unknown. GLP-1R expression within ventricular myocytes has not been consistently shown (Baggio et al, 2018; Taktaz et al, 2024), though emerging evidence suggesting that GLP-1RA effects extend beyond glycemic regulation. Genetic cardiomyocyte-specific disruption of *Glp1r* in mice failed to prevent cardioprotection by GLP-1RAs in the LAD ligation model of ischemic cardiomyopathy (Ussher et al, 2014). By contrast, in vitro GLP-1R agonism in non-cardiac tissue mitigates hyperglycemia-induced hypomethylation at the NF-κB and SOD2 promoters in human aortic endothelial cells, potentially encoding its anti-inflammatory and redox-stabilizing effects (Scisciola et al, 2023). Additionally, endopeptidase-generated GLP-1 metabolites promote PKA-mediated oxidative cytoprotection within the coronary endothelium and attenuate ischemic injury in vivo (Siraj et al, 2020). Glycemic response to GLP-1RA’s is influenced by epigenetic variation, with hypomethylation of the *VTRNA2-1* promoter in peripheral blood correlating with significantly reduced therapeutic efficacy, particularly in individuals carrying the rs2346018 allele (Lin et al, 2021). Therefore, while incretin mimetics deliver measurable cardiovascular benefits, further investigation is necessary to understand their longer-term impact on epigenetic regulation and metabolic remodeling in the heart.

### β-adrenergic receptor blockers and activators (β-ARB and β-ARA)

Among the oldest guideline-directed pharmacologic managements of heart failure are β-ARBs based on their established survival benefit (Packer et al, 1996; Bristow, 2011). And yet, despite clear clinical evidence supporting their implementation, the exact mechanism of β-ARB mediated cardioprotection remains incompletely understood. β adrenergic signaling in cardiomyocytes is well-characterized, but insights gained from adipocytes may offer additional perspectives. Catecholamine-induced lipolysis requires PKA and represents a well-established adaptive short-term mechanism to maintain body homeostasis and energy expenditure (Yang and Mottillo, 2020). We introduced the concept that intact lipolysis maintains cardiac function by counteracting the cardiotoxic production of anaplerotic metabolites of glycolysis (Backs et al, 2011; Jebessa et al, 2019; Lehmann et al, 2018). From this perspective, β-ARBs may potentiate the coupling of β-adrenergic receptors to PKA-dependent lipolysis (Zheng et al, 2005), whereby short-term β-adrenergic activation might paradoxically exert cardioprotection (Fig. 3). It is therefore fitting that selective β3-AR agonists, which also induce lipolysis in adipocytes, have entered clinical trials to improve diastolic performance in HFpEF (Pouleur et al, 2018; Balligand, 2013). Despite this mechanistic justification, clinical trials have not yet proven benefit of β3 agonism in cardiomyopathy. Among these negative trials, the SPHERE-HF was a randomized, double-blind, placebo-controlled phase II trial that measured the effects of β3 agonist mirabegron in patients with HFpEF, only to discover no difference in pulmonary vascular resistance (primary outcome), or right ventricular function (secondary outcome) (García-Álvarez et al, 2023). Thus, additional studies are needed to identify a translational application for the robust preclinical evidence supporting β3 agonism in cardiomyopathy. Further studies using conditional murine knockout alleles of β-adrenergic receptor subtypes (β1-, β2- and β3-adrenergic receptors) would help to distinguish the sub-type specific metabolic consequences of their inhibition and enable the development of more specific—and effective—therapeutic molecules. If proven correct, this perspective would challenge a prevailing viewpoint that β-ARBs confer cardioprotection via direct effect of catecholamines on calcium handling, and instead by exerting their effect on intermediary metabolic circuits.

## Targeting chromatin-modifying enzymes in cardiovascular and metabolic disease

The therapeutic targeting of chromatin-modifying enzymes is rapidly gaining traction in cardiovascular research, driven by growing recognition that their epigenetic and non-epigenetic mechanisms contribute to both the onset and progression of heart failure (Table 3). Regardless of the urgent need to identify and quantify the relative contributions of the direct versus indirect epigenetic targets within the metabo-epigenetic circuitries, the great potential of inhibiting these enzymes in metabolically driven heart disease must not be overlooked. Among these, histone deacetylase inhibition (HDACi) has shown promising cardioprotective effects in preclinical models, while ongoing development of bromodomain and extra-terminal domain (BET) and EZH2 inhibitors further expands the landscape of drug development pipelines, although translation of these therapies into the clinic remains challenged by issues of specificity, systemic toxicity, and unclear long-term efficacy in humans. Although lifestyle interventions such as exercise, weight loss, and dietary modification have been shown to influence the cardiac epigenome, these interventions engage a complex interplay of cellular signals that each warrant a dedicated in-depth discussion that falls beyond the scope of this review.

**Table 3:** Investigational epigenetic therapies.

Drug/compound	Epigenetic target	Mechanism of action	Clinical application	FDA status	Reference
Vorinistat (SAHA)	pan-HDACi (Class I/II)	↓ LVH and fibrosis	Pressure overload HF	Preclinical (mouse)	(Eaton et al, 2022)
SK-7041					(Kee et al, 2005)
Givinostat			Duchenne muscular dystrophy, Pressure overload HF	Phase IV (non-cardiac)	(Travers et al, 2021)
SRT2104	SIRT1	SIRT1 Agonist	Arterial stiffness, ventricular arrhythmias	Phase II	(Venkatasubramanian et al, 2016)
Resveratrol	SIRT1 (Class III)	Restores mitochondrial OXPHOS	Aging-related cardiac dysfunction	Preclinical (mouse)	(Sung et al, 2015)
TN-301 (TYA-018)	HDAC6	↓ HDAC6, ↓ LVH, fibrosis, and mitochondrial energy production	Cardiometabolic HFpEF	Phase Ib	(Ranjbarvaziri et al, 2024)
Sulforaphane	HDAC/DNMT	Activates Nrf2 antioxidant pathway	HFpEF, doxorubicin cardiomyopathy	Preclinical, Phase II (NCT05408559)	(Bose et al, 2018; Ma et al, 2018)
CDR132L	miR-132	Inhibits miR-132, de-represses FoxO3, ↓ LVH and fibrosis	Chronic heart failure, Myocardial infarction	Phase II HF-REVERT (NCT05350969)	(Bauersachs et al, 2024)
AntimiR-21	microRNA-21	Inhibition of miR-21,	Myocardial Infarction, Ischemia-reperfusion injury	Preclinical (pig)	(Hinkel et al, 2020)
AntimiR-29	microRNA-29	Inhibition of miR-29, De-repression of Wnt signaling	Pathological cardiac remodeling and fibrosis	Preclinical (mouse)	(Sassi et al, 2017)
LNA-antimiR-208a	micoRNA-208a	Prevents pathological myosin switching and hypertrophy	HFrEF	Preclinical (rat)	(Montgomery et al, 2011)
JQ1	BET proteins	Nonselective BET inhibition, ↓ inflammatory and profibrotic myocardial genes	HFrEF, Pulmonary arterial hypertension	Preclinical	(Duan et al, 2017)
GSK126	EZH2	Inhibits PRC2, ↓ CpG methylation	Myocardial infarction, HCM	Preclinical	(Aziz et al, 2023)
^a^Apabetalone (RVX-208)	BET proteins (BRD2/BRD3)	Selective bromodomain 2 inhibitor, ↓ inflammatory and profibrotic myocardial gene	MACE in type 2 diabetes after ACS	Phase III BETonMACE trial (NCT02586155)	(Ray et al, 2020)
^b^Decitabine ^b^Azacitidine	DNMTs	Hypomethylate and suppress fibrotic and hypertrophic genes	Pressure overload HF	Preclinical (rat)	(Stenzig et al, 2018)

The following table lists the drug or compound known to influence a chromatin-modifying enzyme with proposed applications in heart failure treatment.

*HDACi* histone deacetylase inhibitor, *LVH* left ventricular hypertrophy, *HF* heart failure, *HFpEF* heart failure with preserved ejection fraction, *OXPHOS* oxidative phosphorylation, *HCM* hypertrophic cardiomyopathy, *PRC2* polycomb repressor complex 2.

^a^Phase III trial failed to demonstrate clinical benefit.

^b^Causes reversible cardiomyopathy.

### HDAC inhibitors (HDACi’s)

The direct targeting of epigenetic regulators, particularly via HDAC inhibition (HDACi), has become an exciting area of ongoing research and development (McKinsey, 2011; Lobera et al, 2013; Di Giorgio et al, 2014; Travers et al, 2021). In the myocardium, HDACs control the activity of many cardiac transcription factors, including GATA4, NFAT and MEF2, and have emerged as promising therapeutic targets in several heart diseases (Wright and Menick, 2016). The use of HDACi has already shown promising results on attenuating cardiac dysfunction and fibrosis (Wang et al, 2016).

Despite many efforts to advance precise control of enzymatic HDAC inhibition, panHDACi’s (e.g., suberoylanilide hydroxamic acid, SAHA) have already been shown to demonstrate therapeutic benefits in preclinical hypertensive HFpEF and ischemic heart disease models (Wallner et al, 2020; Eaton et al, 2022; Travers et al, 2021; Jeong et al, 2018; Wang et al, 2016) but the underlying specific HDAC has not yet been clearly identified. As reviewed above class IIb HDAC inhibitors await clinical testing in HFpEF patients. PanHDACis also inhibit class IIa HDAC such as HDAC4 that also have been shown to specifically regulate cardiac metabolism via its non-enzymatic domain. At this point, we wish to emphasize the strong likelihood that the enzymatic and non-enzymatic actions of HDAC4 are closely interconnected in their biological function. Unpublished data from our group provide proof of concept of enzymatic class IIa HDAC inhibition as a potential new HFpEF therapy. Thus, it remains to be seen whether a subclass or isoform-specific HDAC inhibition approach might be translated to successful clinical trials. Targeted class IIa and class IIb HDAC inhibitors must first be optimized and then be tested in patients to obtain definitive answers for this question. An additional challenge will be the identification and stratification of patients that may derive the most benefit of class IIa and/or class IIb HDAC inhibitors.

However, the effects of HDACi—in particular class I HDACs—on non-cardiac tissues are widespread, as evidenced by their clinical usefulness as adjuvant anti-cancer therapies (Terranova-Barberio et al, 2017). Whether these concerns are justified for the inhibition of class IIa and class IIb HDACs remains to be tested in clinical trials. However—as reviewed above—class IIa HDACs in the myocardium have been shown to control the activity of many cardiac transcription factors, including GATA4, NFAT and MEF2, by a non-enzymatic mode of action, and have thus emerged as promising therapeutic targets in heart disease (Kim et al, 2016; Wright and Menick, 2016; Lehmann et al, 2013). Augmenting the non-enzymatic functions of class IIa HDACs may allow more precise control than enzymatic inhibition.

### BET inhibitors

JQ1, a selective inhibitor of bromodomain and extra-terminal (BET) proteins such as BRD4, has demonstrated cardioprotective effects in preclinical models of heart disease. In murine models of pressure overload and myocardial infarction, JQ1 reduced cardiac hypertrophy, fibrosis, and inflammation while preserving ventricular function, primarily through repression of maladaptive gene networks including NF-κB and TGF-β signaling pathways (Duan et al, 2017). Single-cell transcriptomics have shown that JQ1 reprograms disease-associated cardiac fibroblasts and modulates endothelial and myeloid cell states, contributing to its broad anti-remodeling effects (Alexanian et al, 2021). Importantly, JQ1 spares physiological cardiac growth and attenuates hypertrophy in human iPSC-derived cardiomyocytes, suggesting disease-selective efficacy (Duan et al, 2017). However, clinical translation has proven difficult owing to its limited bioavailability and bioequivalent molecules failing to demonstrate clinical efficacy. Apabetalone (RVX-208) was a clinically tested BET inhibitor with selectivity for the second bromodomain (BD2), designed to reduce toxicity and off-target effects; however, this therapy failed to lower adverse cardiovascular events in diabetic patients enrolled in the BETonMACE trial despite its proven pre-clinical anti-inflammatory and lipid-modifying properties (Ray et al, 2020). Therefore, ongoing investigations are underway to develop more potent BET inhibitors that still lack the concomitant toxicity and off-target consequences.

### EZH2 inhibitors

Owing to our recent work identifying EZH2 as a possible regulator of cardiac metabolism in patients with ischemic heart failure (Pepin et al, 2019b), the use of FDA-approved EZH2 inhibitors may provide a future benefit for patients with ischemic cardiomyopathy (Fig. 2). Evidence already supports a therapeutic role of small-molecule inhibition of EZH2 as a metabolic mechanism of protection against cellular de-differentiation and, consequently, malignant transformation of non-cardiac tissues (Shi et al, 2017; Duan et al, 2020). It has also been shown that suppressing EZH2 enhances the differentiation of cardiac fibroblasts into beating cardiomyocytes (Hirai and Kikyo, 2014). A preliminary report now supports the role of EZH2/PRC2 inhibitors to normalize lipid metabolism in ischemic cardiomyopathy using a murine model of myocardial infarction (Boel et al, 2023). Therefore, it remains plausible, yet unproven, that the induction of EZH2 moderates a metabolic switch within ischemic cardiomyocytes to produce a resting, non-contractile phenotype that can be reprogrammed for clinical HF recovery.

## Conclusion

The breakthroughs highlighted in this review underscore the interconnected roles of cardiac metabolism and histone-modifying enzymes in the pathogenesis of heart disease. Although incompletely understood, we summarize the current evidence supporting the existence of “metabo-epigenetic circuits” that regulate the heart’s response to both physiologic and pathologic metabolic perturbations. In the process, we feature yet-undefined elements of these circuits that merit further mechanistic exploration. It has become evident that histone-modifying enzymes influence metabolism directly and indirectly. The therapeutic targeting of this metabolic and/or epigenetic machinery, through either lifestyle or pharmacologic interventions, offers a promising arena for translational research. Several existing therapies for heart failure were originally developed for other metabolic indications and later found to confer cardioprotective benefits, implicating heart failure as itself a metabolic disease. Ultimately, leveraging the under-appreciated mechanisms of myocardial gene regulation is likely to enable precision-based “metabo-epigenetic” therapies to address the far-reaching, and seemingly intractable, consequences of heart disease and failure.

### Pending issues

- The fundamental architecture of metabo-epigenetic circuits in heart failure remains incomplete, requiring systematic mapping (i.e., with high-dimensional perturbation and multi-omic profiling) to define the missing nodes and metabolic signals that activate maladaptive gene programs.
- Key mechanistic paradoxes persist, including the divergent consequences of HDAC6 inhibition and the context-dependent effects of histone O-GlcNAcylation, necessitating inducible, dose-dependent, and cell type-specific interrogation studies.
- Translating metabo-epigenetic insights into therapy will require disease-relevant drug discovery platforms to develop selective epigenetic modulators, such as isoform-specific HDAC inhibitors and EZH2 blocking agents.

## Supplementary information

Peer Review File

## Glossary

**ABHD5**: Cofactor for PNPLA2/ATGL-mediated triglyceride lipolysis; regulates lipid droplet turnover and releases HDAC4-NT to influence nuclear gene programs.
**Acetyl-CoA**: Central metabolic intermediate derived from carbohydrates, fatty acids, or acetate; donor substrate for histone acetylation linking metabolism to chromatin regulation.
**AMPK (AMP-activated protein kinase)**: Energy-sensing kinase that phosphorylates metabolic enzymes and transcriptional regulators to restore ATP homeostasis.
**ATGL (PNPLA2)**: Rate-limiting triglyceride lipase catalyzing the first step of lipolysis; mutations cause neutral lipid storage disease with cardiomyopathy.
**BET proteins (e.g., BRD4)**: Bromodomain-containing chromatin readers that bind acetylated histones to regulate transcription; pharmacologic inhibition alters maladaptive cardiac gene programs.
**BDH1/OXCT1**: Ketolytic enzymes catalyzing β-hydroxybutyrate oxidation and succinyl-CoA transfer, respectively; essential for cardiac ketone utilization.
**CAMK2**: Ca^2+^/calmodulin-dependent protein kinase implicated in excitation–contraction coupling and maladaptive hypertrophic signaling.
**Chromatin-modifying enzymes**: Enzymes catalyzing post-translational modifications of histones or covalent DNA modifications that alter chromatin accessibility and transcriptional activity.
**DNMT3A**: De novo DNA methyltransferase establishing CpG methylation patterns; regulates metabolic gene expression in cardiomyocytes.
**D-2-hydroxyglutarate (D-2-HG)**: Oncometabolite generated by mutant IDH1/2; competitive inhibitor of TET dioxygenases and Jumonji histone demethylases.
**EZH2/PRC2**: Histone methyltransferase catalyzing H3K27 trimethylation; represses gene expression and has been implicated in ischemic cardiomyopathy.
**Fatty acid oxidation (FAO)**: Mitochondrial β-oxidation pathway generating acetyl-CoA and NADH; predominant energy source in adult myocardium.
**GLP-1 receptor agonists (GLP-1RA)**: Incretin mimetics with cardioprotective effects; epigenetic influence in non-cardiac tissues includes modulation of promoter methylation.
**HDACs (histone deacetylases)**: Enzyme family removing acetyl groups from histones and non-histone proteins; regulate cardiac gene expression, metabolic substrate preference, and cytoskeletal stability.
**HDAC4-NT**: Proteolytic N-terminal fragment of HDAC4 that represses MEF2 activity and prevents maladaptive glycolytic reprogramming.
**HFrEF/HFpEF**: Clinical heart failure subtypes characterized by reduced vs preserved left ventricular ejection fraction, respectively.
**Hexosamine biosynthetic pathway (HBP)**: Glucose-derived metabolic branch producing UDP-GlcNAc for O-GlcNAc protein modification.
**Histones**: Core nucleosomal proteins subject to post-translational modifications that regulate chromatin structure and gene activity.
**IDH1/IDH2**: Isocitrate dehydrogenases producing α-ketoglutarate; neomorphic mutations generate D-2-HG and disrupt epigenetic regulation.
**Ketone bodies**: Alternative mitochondrial substrates (β-hydroxybutyrate, acetoacetate) oxidized by the heart during stress or nutrient deprivation.
**MEF2**: Myocyte enhancer factor 2; transcription factor regulating muscle gene expression and metabolic programs.
**NAD⁺**: Redox cofactor required for sirtuin deacetylases and metabolic enzyme activity; levels influence mitochondrial function and chromatin regulation.
**NHE-1 (Na⁺/H⁺ exchanger 1)**: Membrane antiporter modulating intracellular pH and Ca^2+^ handling; indirectly inhibited by SGLT2 inhibitors.
**NR4A1**: Nuclear receptor transcription factor regulating glycolytic and HBP genes downstream of MEF2 signaling.
**O-GlcNAcylation**: Reversible attachment of N-acetylglucosamine to serine/threonine residues of proteins; integrates nutrient sensing with transcriptional and signaling pathways.
**OGT/OGA**: Enzymes catalyzing the addition (OGT) and removal (OGA) of O-GlcNAc modifications.
**Oncometabolites**: Metabolic intermediates (e.g., succinate, fumarate, D-2-HG) that inhibit α-ketoglutarate–dependent dioxygenases and alter epigenetic landscapes.
**PPARα/PPARγ**: Nuclear hormone receptors regulating fatty acid oxidation (α) and lipid uptake/storage (γ); central regulators of cardiac substrate preference.
**SGLT2 inhibitors**: Antidiabetic agents that improve cardiovascular outcomes; mechanisms include modulation of HDACs and SIRT1 in cardiac tissue.
**TET enzymes**: Ten-eleven translocation dioxygenases catalyzing DNA demethylation via 5-methylcytosine oxidation.
**UDP-GlcNAc**: End product of the HBP; donor substrate for O-GlcNAcylation of nuclear and cytosolic proteins.
**Warburg effect**: Aerobic glycolysis observed in cancer and stressed myocardium; produces metabolites that influence epigenetic modifiers.

## References

[CR1] Ahamed H, Balegadde AV, Menon S, Menon R, Ramachandran A, Mathew N, Natarajan KU, Nair IR, Kannan R, Shankar M et al (2020) Phenotypic expression and clinical outcomes in a South Asian PRKAG2 cardiomyopathy cohort. Sci Rep 10:20610 33244021 10.1038/s41598-020-77124-9 PMC7691361

[CR2] Akbay EA, Moslehi J, Christensen CL, Saha S, Tchaicha JH, Ramkissoon SH, Stewart KM, Carretero J, Kikuchi E, Zhang H et al (2014) D-2-hydroxyglutarate produced by mutant IDH2 causes cardiomyopathy and neurodegeneration in mice. Genes Dev 28:479–490 24589777 10.1101/gad.231233.113 PMC3950345

[CR3] Alexanian M, Przytycki PF, Micheletti R, Padmanabhan A, Ye L, Travers JG, Gonzalez-Teran B, Silva AC, Duan Q, Ranade SS et al (2021) A transcriptional switch governs fibroblast activation in heart disease. Nature 595:438–443 34163071 10.1038/s41586-021-03674-1 PMC8341289

[CR4] Allen DW, Wyman JrJ, Smith CA (1953) The oxygen equilibrium of fetal and adult human hemoglobin. J Biol Chem 203:81–87 13069489

[CR5] Alpert MA, Lavie CJ, Agrawal H, Aggarwal KB, Kumar SA (2014) Obesity and heart failure: epidemiology, pathophysiology, clinical manifestations, and management. Transl Res 164:345–356 24814682 10.1016/j.trsl.2014.04.010

[CR6] Aminian A, Zajichek A, Arterburn DE, Wolski KE, Brethauer SA, Schauer PR, Kattan MW, Nissen SE (2019) Association of metabolic surgery with major adverse cardiovascular outcomes in patients with type 2 diabetes and obesity. JAMA 322:1271–1282 10.1001/jama.2019.14231 PMC6724187 31475297

[CR7] Anker SD, Butler J, Filippatos G, Ferreira JP, Bocchi E, Böhm M, Brunner-La Rocca H-P, Choi D-J, Chopra V, Chuquiure-Valenzuela E et al (2021) Empagliflozin in heart failure with a preserved ejection fraction. N Engl J Med 385:1451–1461 34449189 10.1056/NEJMoa2107038

[CR8] Antonicka H, Mattman A, Carlson CG, Glerum DM, Hoffbuhr KC, Leary SC, Kennaway NG, Shoubridge EA (2002) Mutations in COX15 produce a defect in the mitochondrial heme biosynthetic pathway, causing early-onset fatal hypertrophic cardiomyopathy. Am J Hum Genet 72:101–114 12474143 10.1086/345489 PMC378614

[CR9] Aubert G, Martin OJ, Horton JL, Lai L, Vega RB, Leone TC, Koves T, Gardell SJ, Kruger M, Hoppel CL et al (2016) The failing heart relies on ketone bodies as a fuel. Circulation 133:698–705 26819376 10.1161/CIRCULATIONAHA.115.017355 PMC4766035

[CR10] Aziz S, Yalan L, Raza MA, Lemin J, Akram HMB, Zhao W (2023) GSK126 an inhibitor of epigenetic regulator EZH2 suppresses cardiac fibrosis by regulating the EZH2-PAX6-CXCL10 pathway. Biochem Cell Biol 101:87–100 36469862 10.1139/bcb-2022-0224

[CR11] Baartscheer A, Schumacher CA, Wüst RCI, Fiolet JWT, Stienen GJM, Coronel R, Zuurbier CJ (2016) Empagliflozin decreases myocardial cytoplasmic Na(+) through inhibition of the cardiac Na(+)/H(+) exchanger in rats and rabbits. Diabetologia 60:568–573 27752710 10.1007/s00125-016-4134-x PMC6518059

[CR12] Backs J, Song K, Bezprozvannaya S, Chang S, Olson EN (2006) CaM kinase II selectively signals to histone deacetylase 4 during cardiomyocyte hypertrophy. J Clin Invest 116:1853–1864 16767219 10.1172/JCI27438 PMC1474817

[CR13] Backs J, Worst BC, Lehmann LH, Patrick DM, Jebessa Z, Kreusser MM, Sun Q, Chen L, Heft C, Katus HA et al (2011) Selective repression of MEF2 activity by PKA-dependent proteolysis of HDAC4. J Cell Biol 195:403–415 22042619 10.1083/jcb.201105063 PMC3206346

[CR14] Baggio LL, Yusta B, Mulvihill EE, Cao X, Streutker CJ, Butany J, Cappola TP, Margulies KB, Drucker DJ (2018) GLP-1 receptor expression within the human heart. Endocrinology 159:1570–1584 29444223 10.1210/en.2018-00004 PMC5939638

[CR15] Balligand JL (2013) Beta3-adrenoreceptors in cardiovasular diseases: new roles for an “old” receptor. Curr Drug Deliv 10:64–66 22998044 10.2174/1567201811310010011

[CR16] Bauersachs J, Solomon SD, Anker SD, Antorrena-Miranda I, Batkai S, Viereck J, Rump S, Filippatos G, Granzer U, Ponikowski P et al (2024) Efficacy and safety of CDR132L in patients with reduced left ventricular ejection fraction after myocardial infarction: Rationale and design of the HF-REVERT trial. Eur J Heart Fail 26:674–682 38269451 10.1002/ejhf.3139

[CR17] Bird A (2007) Perceptions of epigenetics. Nature 447:396–398 17522671 10.1038/nature05913

[CR18] Bishop SP, Altschuld RA (1970) Increased glycolytic metabolism in cardiac hypertrophy and congestive failure. Am J Physiol 218:153–159 4243400 10.1152/ajplegacy.1970.218.1.153

[CR19] Boel H, Robillard F, Renet S, Fraineau S, Ruiz M (2023) EZH2 epigenetic enzyme inhibition improves the lipid metabolic disturbances and preserves cardiac function in a mouse model of myocardial infarction. Arch Cardiovasc Dis Suppl 15:239–240

[CR20] Bose C, Awasthi S, Sharma R, Beneš H, Hauer-Jensen M, Boerma M, Singh SP (2018) Sulforaphane potentiates anticancer effects of doxorubicin and attenuates its cardiotoxicity in a breast cancer model. PLoS ONE 13:e0193918 29518137 10.1371/journal.pone.0193918 PMC5843244

[CR21] Boyer JK, Thanigaraj S, Schechtman KB, Perez JE (2004) Prevalence of ventricular diastolic dysfunction in asymptomatic, normotensive patients with diabetes mellitus. Am J Cardiol 93:870–875 15050491 10.1016/j.amjcard.2003.12.026

[CR22] Brahma MK, Ha C-M, Pepin ME, Mia S, Sun Z, Chatham JC, Habegger KM, Abel ED, Paterson AJ, Young ME et al (2020) Increased glucose availability attenuates myocardial ketone body utilization. J Am Heart Assoc 9:e013039 32750298 10.1161/JAHA.119.013039 PMC7792234

[CR23] Braunwald E (1997) Shattuck lecture-cardiovascular medicine at the turn of the millennium: triumphs, concerns, and opportunities. N Engl J Med 337:1360–1369 9358131 10.1056/NEJM199711063371906

[CR24] Bristow MR (2011) Treatment of chronic heart failure with beta-adrenergic receptor antagonists: a convergence of receptor pharmacology and clinical cardiology. Circ Res 109:1176–1194 22034480 10.1161/CIRCRESAHA.111.245092

[CR25] Burattini M, Pisano A, Medeghini V, Rossi S, Luciani GB, D’Amati G, Miragoli M (2024) MELAS syndrome: insights into cardiac mechanics. Vasc Pharm 155:107303

[CR26] Burke MA, Cook SA, Seidman JG, Seidman CE (2016) Clinical and mechanistic insights into the genetics of cardiomyopathy. J Am Coll Cardiol 68:2871–2886 28007147 10.1016/j.jacc.2016.08.079 PMC5843375

[CR27] Catteruccia M, Sauchelli D, Della Marca G, Primiano G, Cuccagna C, Bernardo D, Leo M, Camporeale A, Sanna T, Cianfoni A et al (2015) “Myo-cardiomyopathy” is commonly associated with the A8344G “MERRF” mutation. J Neurol 262:701–710 25559684 10.1007/s00415-014-7632-0

[CR28] Chang S, McKinsey TA, Zhang CL, Richardson JA, Hill JA, Olson EN (2004) Histone deacetylases 5 and 9 govern responsiveness of the heart to a subset of stress signals and play redundant roles in heart development. Mol Cell Biol 24:8467–8476 15367668 10.1128/MCB.24.19.8467-8476.2004 PMC516756

[CR29] Chen J, Chen S, Zhang B, Liu J (2021) SIRT3 as a potential therapeutic target for heart failure. Pharm Res 165:105432 10.1016/j.phrs.2021.105432 33508434

[CR30] Chen T, Liu J, Li N, Wang S, Liu H, Li J, Zhang Y, Bu P (2015) Mouse SIRT3 attenuates hypertrophy-related lipid accumulation in the heart through the deacetylation of LCAD. PLoS ONE 10:e0118909 25748450 10.1371/journal.pone.0118909 PMC4351969

[CR31] Chen Z, Gong L, Zhang P, Li Y, Liu B, Zhang L, Zhuang J, Xiao D (2019) Epigenetic down-regulation of Sirt 1 via DNA methylation and oxidative stress signaling contributes to the gestational diabetes mellitus-induced fetal programming of heart ischemia-sensitive phenotype in late life. Int J Biol Sci 15:1240–1251 31223283 10.7150/ijbs.33044 PMC6567811

[CR32] Chowdhury R, Yeoh KK, Tian YM, Hillringhaus L, Bagg EA, Rose NR, Leung IK, Li XS, Woon EC, Yang M et al (2011) The oncometabolite 2-hydroxyglutarate inhibits histone lysine demethylases. EMBO Rep 12:463–469 21460794 10.1038/embor.2011.43 PMC3090014

[CR33] Chu Y, Jiang M, Wu N, Xu B, Li W, Liu H, Su S, Shi Y, Liu H, Gao X et al (2020) O-GlcNAcylation of SIX1 enhances its stability and promotes Hepatocellular Carcinoma Proliferation. Theranostics 10:9830–9842 32863962 10.7150/thno.45161 PMC7449927

[CR34] Costantino S, Mohammed SA, Ambrosini S, Paneni F (2019) Epigenetic processing in cardiometabolic disease. Atherosclerosis 281:150–158 30290963 10.1016/j.atherosclerosis.2018.09.029

[CR35] Das BB, Hernandez LE, Jayakar P, Chatfield KC, Chrisant M (2019) Novel loss of function in the AGK gene: rare cause of end-stage heart failure. JACC Case Rep 1:11–16 34316732 10.1016/j.jaccas.2019.05.007 PMC8288697

[CR36] Dei Cas A, Khan SS, Butler J, Mentz RJ, Bonow RO, Avogaro A, Tschoepe D, Doehner W, Greene SJ, Senni M et al (2015) Impact of diabetes on epidemiology, treatment, and outcomes of patients with heart failure. JACC Heart Fail 3:136–145 25660838 10.1016/j.jchf.2014.08.004

[CR37] Dewulf JP, Barrea C, Vincent M-F, De Laet C, Van Coster R, Seneca S, Marie S, Nassogne M-C (2016) Evidence of a wide spectrum of cardiac involvement due to ACAD9 mutations: report on nine patients. Mol Genet Metab 118:185–189 27233227 10.1016/j.ymgme.2016.05.005

[CR38] Di Giorgio E, Gagliostro E, Brancolini C (2014) Selective class IIa HDAC inhibitors: myth or reality. Cell Mol Life Sci 72:73–86 25189628 10.1007/s00018-014-1727-8 PMC11113455

[CR39] Doenst T, Nguyen TD, Abel ED (2013) Cardiac metabolism in heart failure: implications beyond ATP production. Circ Res 113:709–724 23989714 10.1161/CIRCRESAHA.113.300376 PMC3896379

[CR40] Dorn 2ndGW (2013) Mitochondrial dynamics in heart disease. Biochim Biophys Acta 1833:233–241 22450031 10.1016/j.bbamcr.2012.03.008 PMC3390438

[CR41] Doubilet PM, Benson CB (1995) Embryonic heart rate in the early first trimester: what rate is normal?. J Ultrasound Med 14:431–434 7658510 10.7863/jum.1995.14.6.431

[CR42] Duan Q, McMahon S, Anand P, Shah H, Thomas S, Salunga HT, Huang Y, Zhang R, Sahadevan A, Lemieux ME, et al (2017) BET bromodomain inhibition suppresses innate inflammatory and profibrotic transcriptional networks in heart failure. Sci Transl Med 9:eaah5084 10.1126/scitranslmed.aah5084 PMC5544253 28515341

[CR43] Duan R, Du W, Guo W (2020) EZH2: a novel target for cancer treatment. J Hematol Oncol 13:104 32723346 10.1186/s13045-020-00937-8 PMC7385862

[CR44] Dubois-Deruy E, Belliard A, Mulder P, Bouvet M, Smet-Nocca C, Janel S, Lafont F, Beseme O, Amouyel P, Richard V et al (2015) Interplay between troponin T phosphorylation and O-N-acetylglucosaminylation in ischaemic heart failure. Cardiovasc Res 107:56–65 25916824 10.1093/cvr/cvv136

[CR45] Eaton DM, Martin TG, Kasa M, Djalinac N, Ljubojevic-Holzer S, Von Lewinski D, Pöttler M, Kampaengsri T, Krumphuber A, Scharer K et al (2022) HDAC inhibition regulates cardiac function by increasing myofilament calcium sensitivity and decreasing diastolic tension. Pharmaceutics 14:1509 35890404 10.3390/pharmaceutics14071509 PMC9323146

[CR46] Etchegaray JP, Mostoslavsky R (2016) Interplay between metabolism and epigenetics: a nuclear adaptation to environmental changes. Mol Cell 62:695–711 27259202 10.1016/j.molcel.2016.05.029 PMC4893201

[CR47] Fathi A, Vickneson K, Singh JS (2021) SGLT2-inhibitors; more than just glycosuria and diuresis. Heart Fail Rev 26:623–642 10.1007/s10741-020-10038-w PMC8024235 33274396

[CR48] Fischer J, Lefevre C, Morava E, Mussini JM, Laforet P, Negre-Salvayre A, Lathrop M, Salvayre R (2007) The gene encoding adipose triglyceride lipase (PNPLA2) is mutated in neutral lipid storage disease with myopathy. Nat Genet 39:28–30 17187067 10.1038/ng1951

[CR49] Fischle W, Dequiedt F, Hendzel MJ, Guenther MG, Lazar MA, Voelter W, Verdin E (2002) Enzymatic activity associated with class II HDACs is dependent on a multiprotein complex containing HDAC3 and SMRT/N-CoR. Mol Cell 9:45–57 11804585 10.1016/s1097-2765(01)00429-4

[CR50] Fisher DJ (1984) Oxygenation and metabolism in the developing heart. Semin Perinatol 8:217–225 6234661

[CR51] Fitchett D, Zinman B, Wanner C, Lachin JM, Hantel S, Salsali A, Johansen OE, Woerle HJ, Broedl UC, Inzucchi SE et al (2016) Heart failure outcomes with empagliflozin in patients with type 2 diabetes at high cardiovascular risk: results of the EMPA-REG OUTCOME(R) trial. Eur Heart J 37:1526–1534 26819227 10.1093/eurheartj/ehv728 PMC4872285

[CR52] Forelli N, Eaton D, Patel J, Bowman CE, Kawakami R, Kuznetsov IA, Li K, Brady C, Bedi K, Yang Y et al (2024) SGLT2 inhibitors activate pantothenate kinase in the human heart. Preprint at 10.1101/2024.07.26.605401

[CR53] García-Álvarez A, Blanco I, García-Lunar I, Jordà P, Rodriguez-Arias JJ, Fernández-Friera L, Zegri I, Nuche J, Gomez-Bueno M, Prat S et al (2023) β3 adrenergic agonist treatment in chronic pulmonary hypertension associated with heart failure (SPHERE-HF): a double blind, placebo-controlled, randomized clinical trial. Eur J Heart Fail 25:373–385 36404400 10.1002/ejhf.2745

[CR54] Gaston G, Babcock S, Ryals R, Elizondo G, DeVine T, Wafai D, Packwood W, Holden S, Raber J, Lindner JR et al (2023) A G1528C Hadha knock-in mouse model recapitulates aspects of human clinical phenotypes for long-chain 3-hydroxyacyl-CoA dehydrogenase deficiency. Commun Biol 6:890 37644104 10.1038/s42003-023-05268-1 PMC10465608

[CR55] Gerstein HC, Colhoun HM, Dagenais GR, Diaz R, Lakshmanan M, Pais P, Probstfield J, Riesmeyer JS, Riddle MC, Rydén L et al (2019) Dulaglutide and cardiovascular outcomes in type 2 diabetes (REWIND): a double-blind, randomised placebo-controlled trial. Lancet 394:121–130 31189511 10.1016/S0140-6736(19)31149-3

[CR56] Gorski PA, Jang SP, Jeong D, Lee A, Lee P, Oh JG, Chepurko V, Yang DK, Kwak TH, Eom SH et al (2019) Role of SIRT1 in modulating acetylation of the sarco-endoplasmic reticulum Ca2+-ATPase in heart failure. Circ Res 124:e63–e80 30786847 10.1161/CIRCRESAHA.118.313865 PMC6483854

[CR57] Guellich A, Damy T, Lecarpentier Y, Conti M, Claes V, Samuel JL, Quillard J, Hebert JL, Pineau T, Coirault C (2007) Role of oxidative stress in cardiac dysfunction of PPARalpha-/- mice. Am J Physiol Heart Circ Physiol 293:H93–H102 17369471 10.1152/ajpheart.00037.2007

[CR58] Haemmerle G, Lass A, Zimmermann R, Gorkiewicz G, Meyer C, Rozman J, Heldmaier G, Maier R, Theussl C, Eder S et al (2006) Defective lipolysis and altered energy metabolism in mice lacking adipose triglyceride lipase. Science 312:734–737 16675698 10.1126/science.1123965

[CR59] Haemmerle G, Moustafa T, Woelkart G, Buttner S, Schmidt A, van de Weijer T, Hesselink M, Jaeger D, Kienesberger PC, Zierler K et al (2011) ATGL-mediated fat catabolism regulates cardiac mitochondrial function via PPAR-alpha and PGC-1. Nat Med 17:1076–1085 21857651 10.1038/nm.2439 PMC3244833

[CR60] Hamirani YS, Kundu BK, Zhong M, McBride A, Li Y, Davogustto GE, Taegtmeyer H, Bourque JM (2016) Noninvasive detection of early metabolic left ventricular remodeling in systemic hypertension. Cardiology 133:157–162 26594908 10.1159/000441276 PMC4677787

[CR61] Helmstadter KG, Ljubojevic-Holzer S, Wood BM, Taheri KD, Sedej S, Erickson JR, Bossuyt J, Bers DM (2021) CaMKII and PKA-dependent phosphorylation co-regulate nuclear localization of HDAC4 in adult cardiomyocytes. Basic Res Cardiol 116:11 33590335 10.1007/s00395-021-00850-2 PMC7884572

[CR62] Hinkel R, Ramanujam D, Kaczmarek V, Howe A, Klett K, Beck C, Dueck A, Thum T, Laugwitz K-L, Maegdefessel L et al (2020) AntimiR-21 prevents myocardial dysfunction in a pig model of ischemia/reperfusion injury. J Am Coll Cardiol 75:1788–1800 32299591 10.1016/j.jacc.2020.02.041

[CR63] Hirai H, Kikyo N (2014) Inhibitors of suppressive histone modification promote direct reprogramming of fibroblasts to cardiomyocyte-like cells. Cardiovasc Res 102:188–190 24477643 10.1093/cvr/cvu023 PMC3958621

[CR64] Holman RR, Bethel MA, Mentz RJ, Thompson VP, Lokhnygina Y, Buse JB, Chan JC, Choi J, Gustavson SM, Iqbal N et al (2017) Effects of once-weekly exenatide on cardiovascular outcomes in type 2 diabetes. N Engl J Med 377:1228–1239 28910237 10.1056/NEJMoa1612917 PMC9792409

[CR65] Hsu A, Duan Q, McMahon S, Huang Y, Wood SA, Gray NS, Wang B, Bruneau BG, Haldar SM (2020) Salt-inducible kinase 1 maintains HDAC7 stability to promote pathologic cardiac remodeling. J Clin Invest 130:2966–2977 32106109 10.1172/JCI133753 PMC7259992

[CR66] Hsu Y-HR, Yogasundaram H, Parajuli N, Valtuille L, Sergi C, Oudit GY (2016) MELAS syndrome and cardiomyopathy: linking mitochondrial function to heart failure pathogenesis. Heart Fail Rev 21:103–116 26712328 10.1007/s10741-015-9524-5

[CR67] Jebessa ZH, Shanmukha KD, Dewenter M, Lehmann LH, Xu C, Schreiter F, Siede D, Gong XM, Worst BC, Federico G et al (2019) The lipid droplet-associated protein ABHD5 protects the heart through proteolysis of HDAC4. Nat Metab 1:1157–1167 31742248 10.1038/s42255-019-0138-4 PMC6861130

[CR68] Jeong MY, Lin YH, Wennersten SA, Demos-Davies KM, Cavasin MA, Mahaffey JH, Monzani V, Saripalli C, Mascagni P, Reece TB et al (2018) Histone deacetylase activity governs diastolic dysfunction through a nongenomic mechanism. Sci Transl Med 10:eaao0144 29437146 10.1126/scitranslmed.aao0144 PMC5908215

[CR210] Jin L, Yuan F, Dai G, Yao Q, Xiang H, Wang L, Xue B, Shan Y, Liu X (2020) Blockage of O-linked GlcNAcylation induces AMPK-dependent autophagy in bladder cancer cells. Cell Mol Biol Lett 25:17 10.1186/s11658-020-00208-x PMC7063793 32174982

[CR69] Joehanes R, Just AC, Marioni RE, Pilling LC, Reynolds LM, Mandaviya PR, Guan W, Xu T, Elks CE, Aslibekyan S et al (2016) Epigenetic signatures of cigarette smoking. Circ Cardiovasc Genet 9:436–447 27651444 10.1161/CIRCGENETICS.116.001506 PMC5267325

[CR70] Karlstaedt A, Zhang X, Vitrac H, Harmancey R, Vasquez H, Wang JH, Goodell MA, Taegtmeyer H (2016) Oncometabolite d-2-hydroxyglutarate impairs alpha-ketoglutarate dehydrogenase and contractile function in rodent heart. Proc Natl Acad Sci USA 113:10436–10441 27582470 10.1073/pnas.1601650113 PMC5027422

[CR71] Kassiotis C, Rajabi M, Taegtmeyer H (2008) Metabolic reserve of the heart: the forgotten link between contraction and coronary flow. Prog Cardiovasc Dis 51:74–88 18634919 10.1016/j.pcad.2007.11.005 PMC3645900

[CR72] Kee HJ, Sohn IS, Nam KI, Park JE, Qian YR, Yin Z, Ahn Y, Jeong MH, Bang Y-J, Kim N et al (2005) Inhibition of histone deacetylation blocks cardiac hypertrophy induced by angiotensin II infusion and aortic banding. Circulation 113:51–59 16380549 10.1161/CIRCULATIONAHA.105.559724

[CR73] Kenny HC, Abel ED (2019) Heart failure in type 2 diabetes mellitus. Circ Res 124:121–141 30605420 10.1161/CIRCRESAHA.118.311371 PMC6447311

[CR74] Khan D, Ara T, Ravi V, Rajagopal R, Tandon H, Parvathy J, Gonzalez EA, Asirvatham-Jeyaraj N, Krishna S, Mishra S et al (2021) SIRT6 transcriptionally regulates fatty acid transport by suppressing PPARγ. Cell Rep 35:109190 34077730 10.1016/j.celrep.2021.109190 PMC8190874

[CR204] Khan D, Sarikhani M, Dasgupta S, Maniyadath B, Pandit AS, Mishra S, Ahamed F, Dubey A, Fathma N, Atreya HS, et al (2018) SIRT6 deacetylase transcriptionally regulates glucose metabolism in heart. J Cell Physiol 233:5478–5489 10.1002/jcp.26434 29319170

[CR75] Kienesberger PC, Pulinilkunnil T, Nagendran J, Young ME, Bogner-Strauss JG, Hackl H, Khadour R, Heydari E, Haemmerle G, Zechner R et al (2013) Early structural and metabolic cardiac remodelling in response to inducible adipose triglyceride lipase ablation. Cardiovasc Res 99:442–451 23708736 10.1093/cvr/cvt124 PMC3718322

[CR76] Kienesberger PC, Pulinilkunnil T, Sung MM, Nagendran J, Haemmerle G, Kershaw EE, Young ME, Light PE, Oudit GY, Zechner R et al (2012) Myocardial ATGL overexpression decreases the reliance on fatty acid oxidation and protects against pressure overload-induced cardiac dysfunction. Mol Cell Biol 32:740–750 22158969 10.1128/MCB.06470-11 PMC3272983

[CR77] Kim SY, Morales CR, Gillette TG, Hill JA (2016) Epigenetic regulation in heart failure. Curr Opin Cardiol 31:255–265 27022893 10.1097/HCO.0000000000000276 PMC4955576

[CR78] Komuro I, Yazaki Y (1993) Control of cardiac gene expression by mechanical stress. Annu Rev Physiol 55:55–75 8466185 10.1146/annurev.ph.55.030193.000415

[CR79] Koutroumpakis E, Jozwik B, Aguilar D, Taegtmeyer H (2020) Strategies of unloading the failing heart from metabolic stress. Am J Med 133:290–296 31520618 10.1016/j.amjmed.2019.08.035 PMC7054139

[CR80] Kronlage M, Dewenter M, Grosso J, Fleming T, Oehl U, Lehmann LH, Falcao-Pires I, Leite-Moreira AF, Volk N, Grone HJ et al (2019) O-GlcNAcylation of histone deacetylase 4 protects the diabetic heart from failure. Circulation 140:580–594 31195810 10.1161/CIRCULATIONAHA.117.031942

[CR81] Kundu BK, Zhong M, Sen S, Davogustto G, Keller SR, Taegtmeyer H (2015) Remodeling of glucose metabolism precedes pressure overload-induced left ventricular hypertrophy: review of a hypothesis. Cardiology 130:211–220 25791172 10.1159/000369782 PMC4394867

[CR82] Lan Y, Banks KM, Pan H, Verma N, Dixon GR, Zhou T, Ding B, Elemento O, Chen S, Huangfu D et al (2021) Stage-specific regulation of DNA methylation by TET enzymes during human cardiac differentiation. Cell Rep 37:110095 34879277 10.1016/j.celrep.2021.110095 PMC11229417

[CR83] Lee DS, Pencina MJ, Benjamin EJ, Wang TJ, Levy D, O’Donnell CJ, Nam BH, Larson MG, D’Agostino RB, Vasan RS (2006) Association of parental heart failure with risk of heart failure in offspring. N Engl J Med 355:138–147 16837677 10.1056/NEJMoa052948

[CR84] Lehmann LH, Jebessa ZH, Kreusser MM, Horsch A, He T, Kronlage M, Dewenter M, Sramek V, Oehl U, Krebs-Haupenthal J et al (2018) A proteolytic fragment of histone deacetylase 4 protects the heart from failure by regulating the hexosamine biosynthetic pathway. Nat Med 24:62–72 29227474 10.1038/nm.4452

[CR85] Lehmann LH, Worst BC, Stanmore DA, Backs J (2013) Histone deacetylase signaling in cardioprotection. Cell Mol Life Sci 71:1673–1690 24310814 10.1007/s00018-013-1516-9 PMC3983897

[CR86] Lejeune S, Roy C, Slimani A, Pasquet A, Vancraeynest D, Vanoverschelde J-L, Gerber BL, Beauloye C, Pouleur A-C (2021) Diabetic phenotype and prognosis of patients with heart failure and preserved ejection fraction in a real life cohort. Cardiovasc Diabetol 20:48 33608002 10.1186/s12933-021-01242-5 PMC7893869

[CR205] Li H, Fan J, Zhao Y, Zhang X, Dai B, Zhan J, Yin Z, Nie X, Fu X-D, Chen C, et al (2019) Nuclear miR-320 mediates diabetes-induced cardiac dysfunction by activating transcription of fatty acid metabolic genes to cause lipotoxicity in the heart. Circ Res 125:1106–1120 10.1161/CIRCRESAHA.119.314898 PMC6903355 31638474

[CR87] Libell EM, Richardson JA, Lutz KL, Ng BY, Mockler SRH, Laubscher KM, Stephan CM, Zimmerman BM, Edens ER, Reinking BE et al (2020) Cardiomyopathy in limb girdle muscular dystrophy R9, FKRP related. Muscle Nerve 62:626–632 32914449 10.1002/mus.27052 PMC7693230

[CR88] Lin C-H, Lee Y-S, Huang Y-Y, Tsai C-N (2021) Methylation status of vault RNA 2-1 promoter is a predictor of glycemic response to glucagon-like peptide-1 analog therapy in type 2 diabetes mellitus. BMJ Open Diabetes Res Care 9 10.1136/bmjdrc-2020-001416 PMC7938984 33674278

[CR89] Lin Y-H, Major JL, Liebner T, Hourani Z, Travers JG, Wennersten SA, Haefner KR, Cavasin MA, Wilson CE, Jeong MY et al (2022) HDAC6 modulates myofibril stiffness and diastolic function of the heart. J Clin Invest 132 10.1172/JCI148333 PMC9106344 35575093

[CR90] Liu T, Wu J, Shi S, Cui B, Xiong F, Yang S, Yan M (2023) Dapagliflozin attenuates cardiac remodeling and dysfunction in rats with β-adrenergic receptor overactivation through restoring calcium handling and suppressing cardiomyocyte apoptosis. Diab Vasc Dis Res 20:14791641231197106 37589258 10.1177/14791641231197106 PMC10437211

[CR91] Liu Z, Zhang Y, Qiu C, Zhu H, Pan S, Jia H, Kang H, Guan G, Hui R, Zhu L et al (2020) Diabetes mellitus exacerbates post-myocardial infarction heart failure by reducing sarcolipin promoter methylation. ESC Heart Fail 7:1935–1948 32525286 10.1002/ehf2.12789 PMC7373908

[CR92] Lobera M, Madauss KP, Pohlhaus DT, Wright QG, Trocha M, Schmidt DR, Baloglu E, Trump RP, Head MS, Hofmann GA et al (2013) Selective class IIa histone deacetylase inhibition via a nonchelating zinc-binding group. Nat Chem Biol 9:319–325 23524983 10.1038/nchembio.1223

[CR93] Loison L (2021) Epigenetic inheritance and evolution: a historian’s perspective. Philos Trans R Soc Lond B Biol Sci 376:20200120 33866812 10.1098/rstb.2020.0120 PMC8059632

[CR94] Lopaschuk GD, Collins-Nakai RL, Itoi T (1992) Developmental changes in energy substrate use by the heart. Cardiovasc Res 26:1172–1180 1288863 10.1093/cvr/26.12.1172

[CR95] Lopaschuk GD, Kelly DP (2008) Signalling in cardiac metabolism. Cardiovasc Res 79:205–207 18515845 10.1093/cvr/cvn134

[CR96] Lopaschuk GD, Verma S (2020) Mechanisms of cardiovascular benefits of sodium glucose co-transporter 2 (SGLT2) inhibitors: a state-of-the-art review. JACC Basic Transl Sci 5:632–644 32613148 10.1016/j.jacbts.2020.02.004 PMC7315190

[CR97] Lother A, Bondareva O, Saadatmand AR, Pollmeier L, Hardtner C, Hilgendorf I, Weichenhan D, Eckstein V, Plass C, Bode C et al (2020) Diabetes changes gene expression but not DNA methylation in cardiac cells. J Mol Cell Cardiol 151:74–87 33197445 10.1016/j.yjmcc.2020.11.004

[CR98] Lunde IG, Aronsen JM, Kvaloy H, Qvigstad E, Sjaastad I, Tonnessen T, Christensen G, Gronning-Wang LM, Carlson CR (2012) Cardiac O-GlcNAc signaling is increased in hypertrophy and heart failure. Physiol Genomics 44:162–172 22128088 10.1152/physiolgenomics.00016.2011

[CR99] Ma H-X, Wu K, Dong F-H, Cai B-K, Wu D, Lu H-Y (2024) Effects of Empagliflozin and Dapagliflozin in alleviating cardiac fibrosis through SIRT6-mediated oxidative stress reduction. Sci Rep 14:30764 39730461 10.1038/s41598-024-80829-w PMC11680569

[CR100] Ma T, Zhu D, Chen D, Zhang Q, Dong H, Wu W, Lu H, Wu G (2018) Sulforaphane, a natural isothiocyanate compound, improves cardiac function and remodeling by inhibiting oxidative stress and inflammation in a rabbit model of chronic heart failure. Med Sci Monit 24:1473–1483 29527002 10.12659/MSM.906123 PMC5859672

[CR101] Maack C, Lehrke M, Backs J, Heinzel FR, Hulot JS, Marx N, Paulus WJ, Rossignol P, Taegtmeyer H, Bauersachs J et al (2018) Heart failure and diabetes: metabolic alterations and therapeutic interventions: a state-of-the-art review from the Translational Research Committee of the Heart Failure Association-European Society of Cardiology. Eur Heart J 39:4243–4254 30295797 10.1093/eurheartj/ehy596 PMC6302261

[CR102] Madsen A, Höppner G, Krause J, Hirt MN, Laufer SD, Schweizer M, Tan WLW, Mosqueira D, Anene-Nzelu CG, Lim I et al (2020) An important role for DNMT3A-mediated DNA methylation in cardiomyocyte metabolism and contractility. Circulation 142:1562–1578 32885664 10.1161/CIRCULATIONAHA.119.044444 PMC7566310

[CR103] Madsen A, Krause J, Höppner G, Hirt MN, Tan WLW, Lim I, Hansen A, Nikolaev VO, Foo RSY, Eschenhagen T et al (2021) Hypertrophic signaling compensates for contractile and metabolic consequences of DNA methyltransferase 3A loss in human cardiomyocytes. J Mol Cell Cardiol 154:115–123 33582159 10.1016/j.yjmcc.2021.02.002

[CR104] Mahmood SS, Wang TJ (2013) The epidemiology of congestive heart failure: the Framingham Heart Study perspective. Glob Heart 8:77–82 23998000 10.1016/j.gheart.2012.12.006 PMC3756692

[CR105] Majeed Y, Halabi N, Madani AY, Engelke R, Bhagwat AM, Abdesselem H, Agha MV, Vakayil M, Courjaret R, Goswami N et al (2021) SIRT1 promotes lipid metabolism and mitochondrial biogenesis in adipocytes and coordinates adipogenesis by targeting key enzymatic pathways. Sci Rep 11:8177 33854178 10.1038/s41598-021-87759-x PMC8046990

[CR106] Mangukiya NP, Kaleem S, Meghana DR, Ishfaq L, Kochhar G, Mathew B, Pulekar S, Lainingwala AC, Parmar MP, Venugopal V (2023) Chanarin-Dorfman syndrome (CDS): A rare lipid metabolism disorder. Cureus 15:e43889 37746493 10.7759/cureus.43889 PMC10515467

[CR107] Mant J, Doust J, Roalfe A, Barton P, Cowie MR, Glasziou P, Mant D, McManus RJ, Holder R, Deeks J et al (2009) Systematic review and individual patient data meta-analysis of diagnosis of heart failure, with modelling of implications of different diagnostic strategies in primary care. Health Technol Assess 13:1–207 19586584 10.3310/hta13320

[CR108] Marso SP, Bain SC, Consoli A, Eliaschewitz FG, Jódar E, Leiter LA, Lingvay I, Rosenstock J, Seufert J, Warren ML et al (2016a) Semaglutide and cardiovascular outcomes in patients with type 2 diabetes. N Engl J Med 375:1834–1844 27633186 10.1056/NEJMoa1607141

[CR109] Marso SP, Daniels GH, Brown-Frandsen K, Kristensen P, Mann JFE, Nauck MA, Nissen SE, Pocock S, Poulter NR, Ravn LS et al (2016b) Liraglutide and cardiovascular outcomes in type 2 diabetes. N Engl J Med 375:311–322 27295427 10.1056/NEJMoa1603827 PMC4985288

[CR110] Marwick TH, Ritchie R, Shaw JE, Kaye D (2018) Implications of underlying mechanisms for the recognition and management of diabetic cardiomyopathy. J Am Coll Cardiol 71:339–351 29348027 10.1016/j.jacc.2017.11.019

[CR111] Mazzarotto F, Tayal U, Buchan RJ, Midwinter W, Wilk A, Whiffin N, Govind R, Mazaika E, de Marvao A, Dawes TJW et al (2020) Reevaluating the genetic contribution of monogenic dilated cardiomyopathy. Circulation 141:387–398 31983221 10.1161/CIRCULATIONAHA.119.037661 PMC7004454

[CR112] McKinsey TA (2011) Therapeutic potential for HDAC inhibitors in the heart. Annu Rev Pharm Toxicol 52:303–319 10.1146/annurev-pharmtox-010611-134712 21942627

[CR113] McKinsey TA, Zhang CL, Lu J, Olson EN (2000) Signal-dependent nuclear export of a histone deacetylase regulates muscle differentiation. Nature 408:106–111 11081517 10.1038/35040593 PMC4459600

[CR114] Montgomery RL, Hullinger TG, Semus HM, Dickinson BA, Seto AG, Lynch JM, Stack C, Latimer PA, Olson EN, van Rooij E (2011) Therapeutic inhibition of miR-208a improves cardiac function and survival during heart failure. Circulation 124:1537–1547 21900086 10.1161/CIRCULATIONAHA.111.030932 PMC3353551

[CR115] Montgomery RL, Potthoff MJ, Haberland M, Qi X, Matsuzaki S, Humphries KM, Richardson JA, Bassel-Duby R, Olson EN (2008) Maintenance of cardiac energy metabolism by histone deacetylase 3 in mice. J Clin Invest 118:3588–3597 18830415 10.1172/JCI35847 PMC2556240

[CR206] Movassagh M, Choy M-K, Goddard M, Bennett MR, Down TA, Foo RS-Y (2010) Differential DNA methylation correlates with differential expression of angiogenic factors in human heart failure. PLoS One 5:e8564 10.1371/journal.pone.0008564 PMC2797324 20084101

[CR116] Murashige D, Jang C, Neinast M, Edwards JJ, Cowan A, Hyman MC, Rabinowitz JD, Frankel DS, Arany Z (2020) Comprehensive quantification of fuel use by the failing and nonfailing human heart. Science 370:364–368 33060364 10.1126/science.abc8861 PMC7871704

[CR117] Muthusamy S, DeMartino AM, Watson LJ, Brittian KR, Zafir A, Dassanayaka S, Hong KU, Jones SP (2014) MicroRNA-539 is up-regulated in failing heart, and suppresses O-GlcNAcase expression. J Biol Chem 289:29665–29676 25183011 10.1074/jbc.M114.578682 PMC4207981

[CR118] Nagendran J, Pulinilkunnil T, Kienesberger PC, Sung MM, Fung D, Febbraio M, Dyck JR (2013) Cardiomyocyte-specific ablation of CD36 improves post-ischemic functional recovery. J Mol Cell Cardiol 63:180–188 23948483 10.1016/j.yjmcc.2013.07.020

[CR119] Neubauer S (2007) The failing heart-an engine out of fuel. N Engl J Med 356:1140–1151 17360992 10.1056/NEJMra063052

[CR120] Nishino I, Fu J, Tanji K, Yamada T, Shimojo S, Koori T, Mora M, Riggs JE, Oh SJ, Koga Y et al (2000) Primary LAMP-2 deficiency causes X-linked vacuolar cardiomyopathy and myopathy (Danon disease). Nature 406:906–910 10972294 10.1038/35022604

[CR121] Oka S, Zhai P, Yamamoto T, Ikeda Y, Byun J, Hsu CP, Sadoshima J (2015) Peroxisome proliferator activated receptor-alpha association with silent information regulator 1 suppresses cardiac fatty acid metabolism in the failing heart. Circ Heart Fail 8:1123–1132 26443578 10.1161/CIRCHEARTFAILURE.115.002216 PMC4651813

[CR122] Packer M, Bristow MR, Cohn JN, Colucci WS, Fowler MB, Gilbert EM, Shusterman NH (1996) The effect of carvedilol on morbidity and mortality in patients with chronic heart failure. U.S. Carvedilol Heart Failure Study Group. N Engl J Med 334:1349–1355 8614419 10.1056/NEJM199605233342101

[CR123] Palomer X, Román-Azcona MS, Pizarro-Delgado J, Planavila A, Villarroya F, Valenzuela-Alcaraz B, Crispi F, Sepúlveda-Martínez Á, Miguel-Escalada I, Ferrer J et al (2020) SIRT3-mediated inhibition of FOS through histone H3 deacetylation prevents cardiac fibrosis and inflammation. Signal Transduct Target Ther 5:14 32296036 10.1038/s41392-020-0114-1 PMC7046732

[CR124] Paluvai H, Shanmukha KD, Tyedmers J, Backs J (2023) Insights into the function of HDAC3 and NCoR1/NCoR2 co-repressor complex in metabolic diseases. Front Mol Biosci 10:1190094 37674539 10.3389/fmolb.2023.1190094 PMC10477789

[CR125] Parini R, Menni F, Garavaglia B, Fesslova V, Melotti D, Massone ML, Lamantea E, Rimoldi M, Vizziello P, Gatti R (1998) Acute, severe cardiomyopathy as main symptom of late-onset very long-chain acyl-coenzyme A dehydrogenase deficiency. Eur J Pediatr 157:992–995 9877038 10.1007/s004310050984

[CR126] Pearen MA, Muscat GE (2010) Minireview: Nuclear hormone receptor 4A signaling: implications for metabolic disease. Mol Endocrinol 24:1891–1903 20392876 10.1210/me.2010-0015 PMC5417389

[CR127] Pennisi EM, Arca M, Bertini E, Bruno C, Cassandrini D, D’Amico A, Garibaldi M, Gragnani F, Maggi L, Massa R et al (2017) Neutral Lipid Storage Diseases: clinical/genetic features and natural history in a large cohort of Italian patients. Orphanet J Rare Dis 12:90 28499397 10.1186/s13023-017-0646-9 PMC5427600

[CR128] Pepin ME, Drakos S, Ha C-M, Tristani-Firouzi M, Selzman CH, Fang JC, Wende AR, Wever-Pinzon O (2019a) DNA methylation reprograms cardiac metabolic gene expression in end-stage human heart failure. Am J Physiol Heart Circ Physiol 317:H674–H684 31298559 10.1152/ajpheart.00016.2019 PMC6843013

[CR129] Pepin ME, Ha C-M, Crossman DK, Litovsky SH, Varambally S, Barchue JP, Pamboukian SV, Diakos NA, Drakos SG, Pogwizd SM et al (2019b) Genome-wide DNA methylation encodes cardiac transcriptional reprogramming in human ischemic heart failure. Lab Invest 99:371–386 30089854 10.1038/s41374-018-0104-x PMC6515060

[CR130] Pepin ME, Konrad PJM, Nazir S, Bazgir F, Maack C, Nickel A, Gorman J, Hohl M, Schreiter F, Dewenter M et al (2025) Mitochondrial NNT promotes diastolic dysfunction in cardiometabolic HFpEF. Circ Res 136:1564–157810.1161/CIRCRESAHA.125.32615440340422

[CR131] Pereyra AS, Hasek LY, Harris KL, Berman AG, Damen FW, Goergen CJ, Ellis JM (2017) Loss of cardiac carnitine palmitoyltransferase 2 results in rapamycin-resistant, acetylation-independent hypertrophy. J Biol Chem 292:18443–18456 28916721 10.1074/jbc.M117.800839 PMC5682957

[CR132] Pouleur AC, Anker S, Brito D, Brosteanu O, Hasenclever D, Casadei B, Edelmann F, Filippatos G, Gruson D, Ikonomidis I et al (2018) Rationale and design of a multicentre, randomized, placebo-controlled trial of mirabegron, a Beta3-adrenergic receptor agonist on left ventricular mass and diastolic function in patients with structural heart disease Beta3-left ventricular hypertrophy (Beta3-LVH). ESC Heart Fail 5:830–841 29932311 10.1002/ehf2.12306 PMC6165933

[CR133] Prola A, Pires Da Silva J, Guilbert A, Lecru L, Piquereau J, Ribeiro M, Mateo P, Gressette M, Fortin D, Boursier C et al (2017) SIRT1 protects the heart from ER stress-induced cell death through eIF2α deacetylation. Cell Death Differ 24:343–356 27911441 10.1038/cdd.2016.138 PMC5299716

[CR134] Ranjbarvaziri S, Zeng A, Wu I, Greer-Short A, Farshidfar F, Budan A, Xu E, Shenwai R, Kozubov M, Li C et al (2024) Targeting HDAC6 to treat heart failure with preserved ejection fraction in mice. Nat Commun 15:1352 38409164 10.1038/s41467-024-45440-7 PMC10897156

[CR135] Rathmell JC, Newgard CB (2009) Biochemistry. A glucose-to-gene link. Science 324:1021–1022 19460991 10.1126/science.1174665 PMC2788238

[CR136] Ray KK, Nicholls SJ, Buhr KA, Ginsberg HN, Johansson JO, Kalantar-Zadeh K, Kulikowski E, Toth PP, Wong N, Sweeney M et al (2020) Effect of apabetalone added to standard therapy on major adverse cardiovascular events in patients with recent acute coronary syndrome and type 2 diabetes: a randomized clinical trial. JAMA 323:1565–1573 32219359 10.1001/jama.2020.3308 PMC7101505

[CR137] Razeghi P, Young ME, Alcorn JL, Moravec CS, Frazier OH, Taegtmeyer H (2001) Metabolic gene expression in fetal and failing human heart. Circulation 104:2923–2931 11739307 10.1161/hc4901.100526

[CR138] Ren F-F, Xie Z-Y, Jiang Y-N, Guan X, Chen Q-Y, Lai T-F, Li L (2022) Dapagliflozin attenuates pressure overload-induced myocardial remodeling in mice via activating SIRT1 and inhibiting endoplasmic reticulum stress. Acta Pharm Sin 43:1721–1732 10.1038/s41401-021-00805-2 PMC9253115 34853445

[CR209] Ren X-P, Wu J, Wang X, Sartor MA, Jones K, Qian J, Nicolaou P, Pritchard TJ, Fan G-C (2009) MicroRNA-320 is involved in the regulation of cardiac ischemia/reperfusion injury by targeting heat-shock protein 20. Circulation 119:2357–2366 10.1161/CIRCULATIONAHA.108.814145 PMC2746735 19380620

[CR139] Ritchie RH, Abel ED (2020) Basic mechanisms of diabetic heart disease. Circ Res 126:1501–1525 32437308 10.1161/CIRCRESAHA.120.315913 PMC7251974

[CR140] Rodeheffer RJ (2011) Hypertension and heart failure: the ALLHAT imperative. Circulation 124:1803–1805 22025634 10.1161/CIRCULATIONAHA.111.059303

[CR141] Rodgers JT, Lerin C, Haas W, Gygi SP, Spiegelman BM, Puigserver P (2005) Nutrient control of glucose homeostasis through a complex of PGC-1alpha and SIRT1. Nature 434:113–118 15744310 10.1038/nature03354

[CR142] Rodrigues B, Cam MC, McNeill JH (1995) Myocardial substrate metabolism: implications for diabetic cardiomyopathy. J Mol Cell Cardiol 27:169–179 7760340 10.1016/s0022-2828(08)80016-8

[CR143] Roger VL (2013) Epidemiology of heart failure. Circ Res 113:646–659 23989710 10.1161/CIRCRESAHA.113.300268 PMC3806290

[CR144] Sano S, Oshima K, Wang Y, MacLauchlan S, Katanasaka Y, Sano M, Zuriaga MA, Yoshiyama M, Goukassian D, Cooper MA et al (2018) Tet2-mediated clonal hematopoiesis accelerates heart failure through a mechanism involving the IL-1β/NLRP3 inflammasome. J Am Coll Cardiol 71:875–886 29471939 10.1016/j.jacc.2017.12.037 PMC5828038

[CR145] Saoura M, Powell CA, Kopajtich R, Alahmad A, Al-Balool HH, Albash B, Alfadhel M, Alston CL, Bertini E, Bonnen PE et al (2019) Mutations in ELAC2 associated with hypertrophic cardiomyopathy impair mitochondrial tRNA 3’-end processing. Hum Mutat 40:1731–1748 31045291 10.1002/humu.23777 PMC6764886

[CR146] Sarma S, Mentz RJ, Kwasny MJ, Fought AJ, Huffman M, Subacius H, Nodari S, Konstam M, Swedberg K, Maggioni AP et al (2013) Association between diabetes mellitus and post-discharge outcomes in patients hospitalized with heart failure: findings from the EVEREST trial. Eur J Heart Fail 15:194–202 23059198 10.1093/eurjhf/hfs153 PMC4176083

[CR147] Sassi Y, Avramopoulos P, Ramanujam D, Grüter L, Werfel S, Giosele S, Brunner A-D, Esfandyari D, Papadopoulou AS, De Strooper B et al (2017) Cardiac myocyte miR-29 promotes pathological remodeling of the heart by activating Wnt signaling. Nat Commun 8: 1614 29158499 10.1038/s41467-017-01737-4 PMC5696364

[CR148] Schuster K, Leeke B, Meier M, Wang Y, Newman T, Burgess S, Horsfield JA (2015) A neural crest origin for cohesinopathy heart defects. Hum Mol Genet 24:7005–7016 26420840 10.1093/hmg/ddv402 PMC4654055

[CR149] Schweiger M, Lass A, Zimmermann R, Eichmann TO, Zechner R (2009) Neutral lipid storage disease: genetic disorders caused by mutations in adipose triglyceride lipase/PNPLA2 or CGI-58/ABHD5. Am J Physiol Endocrinol Metab 297:E289–E296 19401457 10.1152/ajpendo.00099.2009

[CR150] Scisciola L, Taktaz F, Fontanella RA, Pesapane A, Surina, Cataldo V, Ghosh P, Franzese M, Puocci A, Paolisso P et al (2023) Targeting high glucose-induced epigenetic modifications at cardiac level: the role of SGLT2 and SGLT2 inhibitors. Cardiovasc Diabetol 22:24 36732760 10.1186/s12933-023-01754-2 PMC9896756

[CR151] Shah S, Henry A, Roselli C, Lin H, Sveinbjornsson G, Fatemifar G, Hedman AK, Wilk JB, Morley MP, Chaffin MD et al (2020) Genome-wide association and Mendelian randomisation analysis provide insights into the pathogenesis of heart failure. Nat Commun 11:163 31919418 10.1038/s41467-019-13690-5 PMC6952380

[CR152] Shao D, Tian R (2015) Glucose transporters in cardiac metabolism and hypertrophy. Compr Physiol 6:331–351 26756635 10.1002/cphy.c150016 PMC4760112

[CR153] Shi K, Zhang G, Fu H, Li X-M, Gao Y, Shi R, Xu H-Y, Li Y, Guo Y-K, Yang Z-G (2024) Glycemic control and clinical outcomes in diabetic patients with heart failure and reduced ejection fraction: insight from ventricular remodeling using cardiac MRI. Cardiovasc Diabetol 23:148 38685007 10.1186/s12933-024-02243-w PMC11059653

[CR154] Shi X, Tasdogan A, Huang F, Hu Z, Morrison SJ, DeBerardinis RJ (2017) The abundance of metabolites related to protein methylation correlates with the metastatic capacity of human melanoma xenografts. Sci Adv 3:eaao5268 29109980 10.1126/sciadv.aao5268 PMC5665593

[CR155] Simioni C, Nardozza LM, Araujo Junior E, Rolo LC, Zamith M, Caetano AC, Moron AF (2011) Heart stroke volume, cardiac output, and ejection fraction in 265 normal fetus in the second half of gestation assessed by 4D ultrasound using spatio-temporal image correlation. J Matern Fetal Neonatal Med 24:1159–1167 21250911 10.3109/14767058.2010.545921

[CR156] Singla M, Guzman G, Griffin AJ, Bharati S (2007) Cardiomyopathy in multiple Acyl-CoA dehydrogenase deficiency: a clinico-pathological correlation and review of literature. Pediatr Cardiol 29:446–451 17912479 10.1007/s00246-007-9119-6

[CR157] Siraj MA, Mundil D, Beca S, Momen A, Shikatani EA, Afroze T, Sun X, Liu Y, Ghaffari S, Lee W et al (2020) Cardioprotective GLP-1 metabolite prevents ischemic cardiac injury by inhibiting mitochondrial trifunctional protein-α. J Clin Invest 130:1392–1404 31985487 10.1172/JCI99934 PMC7269572

[CR158] Smirnova E, Goldberg EB, Makarova KS, Lin L, Brown WJ, Jackson CL (2006) ATGL has a key role in lipid droplet/adiposome degradation in mammalian cells. EMBO Rep 7:106–113 16239926 10.1038/sj.embor.7400559 PMC1369222

[CR159] Smith NL, Felix JF, Morrison AC, Demissie S, Glazer NL, Loehr LR, Cupples LA, Dehghan A, Lumley T, Rosamond WD et al (2010) Association of genome-wide variation with the risk of incident heart failure in adults of European and African ancestry: a prospective meta-analysis from the cohorts for heart and aging research in genomic epidemiology (CHARGE) consortium. Circ Cardiovasc Genet 3:256–266 20445134 10.1161/CIRCGENETICS.109.895763 PMC3025695

[CR160] Spracklen TF, Kasher PR, Kraus S, Botha TL, Page DJ, Kamuli S, Booi Z, Chin A, Laing N, Keavney BD et al (2021) Identification of a POLG variant in a family with arrhythmogenic cardiomyopathy and left ventricular fibrosis. Circ Genom Precis Med 14:e003138 33276707 10.1161/CIRCGEN.120.003138

[CR161] Stenzig J, Schneeberger Y, Löser A, Peters BS, Schaefer A, Zhao R-R, Ng SL, Höppner G, Geertz B, Hirt MN et al (2018) Pharmacological inhibition of DNA methylation attenuates pressure overload-induced cardiac hypertrophy in rats. J Mol Cell Cardiol 120:53–63 29792884 10.1016/j.yjmcc.2018.05.012

[CR162] Struijk PC, Mathews VJ, Loupas T, Stewart PA, Clark EB, Steegers EA, Wladimiroff JW (2008) Blood pressure estimation in the human fetal descending aorta. Ultrasound Obstet Gynecol 32:673–681 18816497 10.1002/uog.6137

[CR163] Sung MM, Das SK, Levasseur J, Byrne NJ, Fung D, Kim TT, Masson G, Boisvenue J, Soltys C-L, Oudit GY et al (2015) Resveratrol treatment of mice with pressure-overload-induced heart failure improves diastolic function and cardiac energy metabolism. Circ Heart Fail 8:128–137 25394648 10.1161/CIRCHEARTFAILURE.114.001677

[CR164] Svahn J, Laforêt P, Vial C, Streichenberger N, Romero N, Bouchet-Séraphin C, Bruneel A, Dupré T, Seta N, Menassa R et al (2019) Dilated cardiomyopathy and limb-girdle muscular dystrophy-dystroglycanopathy due to novel pathogenic variants in the DPM3 gene. Neuromuscul Disord 29:497–502 31266720 10.1016/j.nmd.2019.05.004

[CR165] Taegtmeyer H, Karlstaedt A, Rees ML, Davogustto G (2017) Oncometabolic Tracks in the Heart. Circ Res 120:267–269 28104766 10.1161/CIRCRESAHA.116.310115 PMC6237182

[CR166] Taegtmeyer H, Sen S, Vela D (2010) Return to the fetal gene program: a suggested metabolic link to gene expression in the heart. Ann N Y Acad Sci 1188:191–198 20201903 10.1111/j.1749-6632.2009.05100.x PMC3625436

[CR167] Taktaz F, Fontanella RA, Scisciola L, Pesapane A, Basilicata MG, Ghosh P, Franzese M, Tortorella G, Puocci A, Vietri MT et al (2024) Bridging the gap between GLP1-receptor agonists and cardiovascular outcomes: evidence for the role of tirzepatide. Cardiovasc Diabetol 23:242 38987789 10.1186/s12933-024-02319-7 PMC11238498

[CR168] Tatekoshi Y, Mahmoodzadeh A, Shapiro JS, Liu M, Bianco GM, Tatekoshi A, Camp SD, De Jesus A, Koleini N, De La Torre S et al (2025) Protein O-GlcNAcylation and hexokinase mitochondrial dissociation drive heart failure with preserved ejection fraction. Cell Metab 37:1584–1600 10.1016/j.cmet.2025.04.001 PMC12221816 40267914

[CR169] Taverna S, Cammarata G, Colomba P, Sciarrino S, Zizzo C, Francofonte D, Zora M, Scalia S, Brando C, Curto AL et al (2020) Pompe disease: pathogenesis, molecular genetics and diagnosis. Aging 12:15856–15874 32745073 10.18632/aging.103794 PMC7467391

[CR170] Terranova-Barberio M, Thomas S, Ali N, Pawlowska N, Park J, Krings G, Rosenblum MD, Budillon A, Munster PN (2017) HDAC inhibition potentiates immunotherapy in triple negative breast cancer. Oncotarget 8:114156–114172 29371976 10.18632/oncotarget.23169 PMC5768393

[CR171] Tong D, Schiattarella GG, Jiang N, Altamirano F, Szweda PA, Elnwasany A, Lee DI, Yoo H, Kass DA, Szweda LI et al (2021) NAD+ repletion reverses heart failure with preserved ejection fraction. Circ Res 128:1629–1641 33882692 10.1161/CIRCRESAHA.120.317046 PMC8159891

[CR172] Travers JG, Wennersten SA, Peña B, Bagchi RA, Smith HE, Hirsch RA, Vanderlinden LA, Lin Y-H, Dobrinskikh E, Demos-Davies KM et al (2021) HDAC inhibition reverses preexisting diastolic dysfunction and blocks covert extracellular matrix remodeling. Circulation 143:1874–1890 33682427 10.1161/CIRCULATIONAHA.120.046462 PMC8884170

[CR173] Umbarawan Y, Kawakami R, Syamsunarno M, Koitabashi N, Obinata H, Yamaguchi A, Hanaoka H, Hishiki T, Hayakawa N, Sunaga H et al (2020) Reduced fatty acid uptake aggravates cardiac contractile dysfunction in streptozotocin-induced diabetic cardiomyopathy. Sci Rep 10:20809 33257783 10.1038/s41598-020-77895-1 PMC7705707

[CR174] Ussher JR, Baggio LL, Campbell JE, Mulvihill EE, Kim M, Kabir MG, Cao X, Baranek BM, Stoffers DA, Seeley RJ et al (2014) Inactivation of the cardiomyocyte glucagon-like peptide-1 receptor (GLP-1R) unmasks cardiomyocyte-independent GLP-1R-mediated cardioprotection. Mol Metab 3:507–517 25061556 10.1016/j.molmet.2014.04.009 PMC4099509

[CR207] Varga ZV, Kupai K, Szűcs G, Gáspár R, Pálóczi J, Faragó N, Zvara A, Puskás LG, Rázga Z, Tiszlavicz L et al (2013) MicroRNA-25- dependent up-regulation of NADPH oxidase 4 (NOX4) mediates hypercholesterolemia-induced oxidative/nitrative stress and subsequent dysfunction in the heart. J Mol Cell Cardiol 62:111–121 10.1016/j.yjmcc.2013.05.009 23722270

[CR175] Venkatasubramanian S, Noh RM, Daga S, Langrish JP, Mills NL, Waterhouse BR, Hoffmann E, Jacobson EW, Lang NN, Frier BM et al (2016) Effects of the small molecule SIRT1 activator, SRT2104 on arterial stiffness in otherwise healthy cigarette smokers and subjects with type 2 diabetes mellitus. Open Heart 3:e000402 27239324 10.1136/openhrt-2016-000402 PMC4879341

[CR176] Waddington CH (2011) The epigenotype. 1942. Int J Epidemiol 41:10–13 22186258 10.1093/ije/dyr184

[CR177] Waitkus MS, Diplas BH, Yan H (2018) Biological role and therapeutic potential of IDH mutations in cancer. Cancer Cell 34:186–195 29805076 10.1016/j.ccell.2018.04.011 PMC6092238

[CR178] Walker MA, Chen H, Yadav A, Ritterhoff J, Villet O, McMillen T, Wang Y, Purcell H, Djukovic D, Raftery D et al (2023) Raising NAD+ level stimulates short-chain dehydrogenase/reductase proteins to alleviate heart failure independent of mitochondrial protein deacetylation. Circulation 148:2038–2057 37965787 10.1161/CIRCULATIONAHA.123.066039 PMC10842390

[CR179] Wallner M, Eaton DM, Berretta RM, Liesinger L, Schittmayer M, Gindlhuber J, Wu J, Jeong MY, Lin YH, Borghetti G et al (2020) HDAC inhibition improves cardiopulmonary function in a feline model of diastolic dysfunction. Sci Transl Med 12: eaay7205 31915304 10.1126/scitranslmed.aay7205 PMC7065257

[CR180] Wang J, Hu X, Jiang H (2016) HDAC inhibition: a novel therapeutic approach for attenuating heart failure by suppressing cardiac remodeling. Int J Cardiol 214:41–42 27057972 10.1016/j.ijcard.2016.03.188

[CR181] Wang S, Wu S, Peng D (2024) Dilated cardiomyopathy caused by mutation of the PNPLA2 gene: a case report and literature review. Front Genet 15:1415156 39119584 10.3389/fgene.2024.1415156 PMC11306180

[CR182] Warburg O (1956) On the origin of cancer cells. Science 123:309–314 13298683 10.1126/science.123.3191.309

[CR183] Weissman D, Dudek J, Sequeira V, Maack C (2024) Fabry disease: cardiac implications and molecular mechanisms. Curr Heart Fail Rep 21:81–100 38289538 10.1007/s11897-024-00645-1 PMC10923975

[CR184] Wellen KE, Hatzivassiliou G, Sachdeva UM, Bui TV, Cross JR, Thompson CB (2009) ATP-citrate lyase links cellular metabolism to histone acetylation. Science 324:1076–1080 19461003 10.1126/science.1164097 PMC2746744

[CR185] Wellen KE, Thompson CB (2012) A two-way street: reciprocal regulation of metabolism and signalling. Nat Rev Mol Cell Biol 13:270–276 22395772 10.1038/nrm3305

[CR186] Wende AR, Abel ED (2010) Lipotoxicity in the heart. Biochim Biophys Acta 1801:311–319 19818871 10.1016/j.bbalip.2009.09.023 PMC2823976

[CR187] Wende AR, Schell JC, Ha C-M, Pepin ME, Khalimonchuk O, Schwertz H, Pereira RO, Brahma MK, Tuinei J, Contreras-Ferrat A et al (2020) Maintaining myocardial glucose utilization in diabetic cardiomyopathy accelerates mitochondrial dysfunction. Diabetes 69:2094–2111 32366681 10.2337/db19-1057 PMC7506832

[CR188] Wolk MJ, Scheidt S, Killip T (1972) Heart failure complicating acute myocardial infarction. Circulation 45:1125–1138 5020802 10.1161/01.cir.45.5.1125

[CR189] Wright LH, Menick DR (2016) A class of their own: exploring the nondeacetylase roles of class IIa HDACs in cardiovascular disease. Am J Physiol Heart Circ Physiol 311:H199–H206 27208161 10.1152/ajpheart.00271.2016 PMC5005290

[CR190] Wu D, Jin J, Qiu Z, Liu D, Luo H (2020) Functional analysis of O-GlcNAcylation in cancer metastasis. Front Oncol 10:585288 33194731 10.3389/fonc.2020.585288 PMC7653022

[CR191] Wu X, Liu H, Brooks A, Xu S, Luo J, Steiner R, Mickelsen DM, Moravec CS, Jeffrey AD, Small EM et al (2022) SIRT6 mitigates heart failure with preserved ejection fraction in diabetes. Circ Res 131:926–943 36278398 10.1161/CIRCRESAHA.121.318988 PMC9669223

[CR192] Xiao M, Yang H, Xu W, Ma S, Lin H, Zhu H, Liu L, Liu Y, Yang C, Xu Y et al (2012) Inhibition of alpha-KG-dependent histone and DNA demethylases by fumarate and succinate that are accumulated in mutations of FH and SDH tumor suppressors. Genes Dev 26:1326–1338 22677546 10.1101/gad.191056.112 PMC3387660

[CR193] Xu W, Yang H, Liu Y, Yang Y, Wang P, Kim SH, Ito S, Yang C, Wang P, Xiao MT et al (2011) Oncometabolite 2-hydroxyglutarate is a competitive inhibitor of alpha-ketoglutarate-dependent dioxygenases. Cancer Cell 19:17–30 21251613 10.1016/j.ccr.2010.12.014 PMC3229304

[CR194] Yan J, Young ME, Cui L, Lopaschuk GD, Liao R, Tian R (2009) Increased glucose uptake and oxidation in mouse hearts prevent high fatty acid oxidation but cause cardiac dysfunction in diet-induced obesity. Circulation 119:2818–2828 19451348 10.1161/CIRCULATIONAHA.108.832915 PMC2765220

[CR195] Yang A, Mottillo EP (2020) Adipocyte lipolysis: from molecular mechanisms of regulation to disease and therapeutics. Biochem J 477:985–1008 32168372 10.1042/BCJ20190468 PMC7187988

[CR196] Yang M, Soga T, Pollard PJ (2013) Oncometabolites: linking altered metabolism with cancer. J Clin Invest 123:3652–3658 23999438 10.1172/JCI67228 PMC3754247

[CR197] Youn HD, Sun L, Prywes R, Liu JO (1999) Apoptosis of T cells mediated by Ca2+-induced release of the transcription factor MEF2. Science 286:790–793 10531067 10.1126/science.286.5440.790

[CR198] Zarich SW, Nesto RW (1989) Diabetic cardiomyopathy. Am Heart J 118:1000–1012 2683698 10.1016/0002-8703(89)90236-6

[CR199] Zhang CL, McKinsey TA, Chang S, Antos CL, Hill JA, Olson EN (2002) Class II histone deacetylases act as signal-responsive repressors of cardiac hypertrophy. Cell 110:479–488 12202037 10.1016/s0092-8674(02)00861-9 PMC4459650

[CR200] Zhang Z, Zheng L, Chen Y, Chen Y, Hou J, Xiao C, Zhu X, Zhao S-M, Xiong J-W (2024) AARS2 ameliorates myocardial ischemia via fine-tuning PKM2-mediated metabolism. eLife 13:RP99670 10.7554/eLife.99670 PMC12080999 40371904

[CR201] Zheng M, Zhu W, Han Q, RP Xiao (2005) Emerging concepts and therapeutic implications of beta-adrenergic receptor subtype signaling. Pharm Ther 108:257–268 10.1016/j.pharmthera.2005.04.006 15979723

[CR202] Zhou R, Barnes K, Gibson S, Fillmore N (2024) Dual-edged role of SIRT1 in energy metabolism and cardiovascular disease. Am J Physiol Heart Circ Physiol 327:H1162–H1173 39269450 10.1152/ajpheart.00001.2024

[CR208] Zhu W-Z, El-Nachef D, Yang X, Ledee D, Olson AK (2019) O-GlcNAc transferase promotes compensated cardiac function and protein kinase A O-GlcNAcylation during early and established pathological hypertrophy from pressure overload. J Am Heart Assoc 8:e011260 10.1161/JAHA.118.011260 PMC6585351 31131693

[CR203] Zhu Q, Zhou L, Yang Z, Lai M, Xie H, Wu L, Xing C, Zhang F, Zheng S (2012) O-GlcNAcylation plays a role in tumor recurrence of hepatocellular carcinoma following liver transplantation. Med Oncol 29:985–993 21461968 10.1007/s12032-011-9912-1

